# Optimizing sustainable blended concrete mixes using deep learning and multi-objective optimization

**DOI:** 10.1038/s41598-025-00943-1

**Published:** 2025-05-10

**Authors:** Rupesh Kumar Tipu, Preeti Rathi, Kartik S. Pandya, Vijay R. Panchal

**Affiliations:** 1 Department of Civil Engineering, School of Engineering & Technology, K. R. Mangalam University, Sohna, Gurugram, 122103 Haryana India; 2 Department of Computer Science & Engineering, School of Engineering & Technology, K. R. Mangalam University, Sohna, Gurugram, 122103 Haryana India; 3 Electrical Engineering Department, Faculty of Engineering and Technology, Parul Institute of Engineering and Technology (PIET), Vadodara, 391760 Gujarat India; 4 M. S. Patel Department of Civil Engineering, Chandubhai S. Patel Institute of Technology (CSPIT), Charotar University of Science and Technology (CHARUSAT), CHARUSAT Campus, Changa, Anand, 388421 Gujarat India

**Keywords:** Green Concrete, Deep Learning, Multi-Objective Optimization, Environmental Impact, Compressive Strength, Engineering, Composites, Mechanical properties

## Abstract

The proposed framework unites deep neural networks (DNNs) together with multi-objective optimization for designing environmentally friendly concrete mixes. A DNN model receives training through a wide dataset which includes multiple mix parameters along with curing conditions for accurate compressive strength prediction. The Bayesian hyperparameter tuning technique produces an optimal network configuration which delivers an average $R^2$ of 0.936 together with an RMSE of 5.71 MPa during 5-fold cross-validation. The Multi-Objective Particle Swarm Optimization (MOPSO) algorithm finds multiple optimal solutions which simultaneously optimize three competing objectives that include strength maximization and cost minimization and cement reduction. The optimized mix designs surpassed 50 MPa compressive strength through cement reduction of up to 25% which led to a total cost reduction of 15% compared to standard mix designs. The analysis of feature importance shows cement content together with concrete age serve as the main factors that affect strength measurements. The integrated data-driven method provides reliable decision-support tools to practitioners who need cost-effective sustainable mix designs through its identification of feasible trade-offs. The proposed methodology delivers new understandings of green concrete technology through optimal proportion discoveries that boost strength and save costs while decreasing environmental impact for direct application in real construction settings.

## Introduction

Growing environmental concerns and depletion of natural resources have prompted a global shift toward sustainable construction practices. Concrete remains the most widely used construction material due to its versatility and structural efficiency^1,2^, but conventional concrete relies heavily on cement-a material whose production is both energy-intensive and carbon-intensive. Cement manufacturing alone accounts for approximately 7% of global CO$_2$ emissions, as reported by the International Energy Agency. Consequently, researchers and practitioners are increasingly exploring ways to reduce concrete’s environmental footprint through improved material selection, mix design, and predictive modeling^3^.

One promising approach to sustainability is the development of “green concrete,” a broad term encompassing mixes that aim to reduce environmental impact through optimized use of materials. In this study, we adopt a working definition of green concrete as concrete that (i) minimizes cement content, (ii) incorporates supplementary cementitious materials (SCMs) such as fly ash and blast furnace slag, and (iii) is optimized to meet performance goals with lower cost and carbon intensity. While some literature associates green concrete exclusively with geopolymer systems, other studies-including^4–6^-have applied the term to Portland-based systems with SCMs, reflecting a broader and widely accepted interpretation. A clarifying note has been added to reflect this distinction.

Traditional concrete mix design methods rely on trial-and-error, which can be time-consuming and inefficient when navigating trade-offs among performance, cost, and sustainability. In contrast, recent advances in computational modeling enable more data-driven decision-making. Machine learning (ML) and deep learning (DL) techniques can uncover complex nonlinear relationships between input features (e.g., ingredient quantities, age, SCM content) and target outputs such as compressive strength or durability metrics. Such tools are increasingly used to support optimized mix design and sustainability assessment.

Recent investigations present multiple approaches for enhancing concrete sustainability by integrating performance-focused optimization with sophisticated computational algorithms. Genetic algorithms, for instance, have been utilized to determine optimal mix proportions that improve mechanical properties and reduce cement demand, although striking a perfect balance between cost and ecological footprint remains challenging^7^. Other studies turn to fuzzy logic and multi-criteria decision-making to handle conflicting design criteria. Moreover, artificial intelligence (AI) has gained substantial momentum in the domain of sustainable concrete research. AI-driven frameworks harness machine learning and multi-objective optimization to optimize mechanical, environmental, and economic outcomes.

Several influential studies illustrate these advances in green concrete mix design. Golafshani, Kim, et al.^4^ utilized artificial intelligence to analyze and optimize recycled aggregate concrete (RAC) mixtures, highlighting AI’s capacity to address both performance and sustainability. Likewise, Faridmehr et al.^6^ developed AI-based optimization schemes to improve ready-mix concrete strength, reduce carbon dioxide emissions, and manage overall costs. Ali et al.^8^ conducted a systematic literature review of AI-driven prediction methods in self-compacting and geopolymer concretes, emphasizing the benefits of data-driven optimization for eco-friendly concrete variants. In another study, Zandifaez et al.^9^ examined the performance of green concrete with recycled aggregates using AI-based predictive modeling and optimization strategies. Their findings show how AI approaches forecast concrete properties and ensure efficient usage of raw materials. Additionally, Liu et al.^5^ introduced a machine learning-based framework that leverages data to optimize the mechanical, economic, and environmental features of sustainable recycled aggregate concrete. These studies collectively highlight the transformative potential of AI and multi-objective optimization for constructing high-performance yet environmentally responsible concrete.

Beyond compressive strength, similar modeling approaches are increasingly found in the study of advanced or specialized applications involving structural members and other mechanical properties. For instance,^10^ proposed using four meta-heuristic algorithms combined with two types of artificial neural networks to optimize the parameters for FRP strength predictions.^11^ applied machine learning regression approaches for predicting the ultimate buckling load of variable-stiffness composite cylinders, demonstrating that deep learning models can yield smaller errors and higher generalization compared with other algorithms.

Moreover,^12^ focused on high-strength steel columns, employing metaheuristic-trained artificial neural networks to accurately estimate the ultimate buckling load. Their work showed that effectively tuned models can achieve high precision (up to 99.8% correlation), underscoring the viability of combining metaheuristics with neural networks. In the realm of concrete mixtures,^13^ investigated M5’ and MARS-based predictive models for self-compacting concrete containing fly ash as a cement replacement, highlighting the importance of rule-based methods and adaptive regression splines for improved prediction accuracy and interpretability. Earlier,^14^ implemented backpropagation neural networks (BPNNs) for predicting the moment-rotation characteristics of semi-rigid connections, demonstrating how neural networks can effectively replace time-consuming finite element analyses for certain structural problems.

Recent research has also expanded into enhanced activation functions.^15^ employed an Exponential Rectified Linear Unit (eReLU) in BPNNs to predict the load-carrying capacity of columns. Their findings revealed improved predictive accuracy and robustness, especially when combined with a comprehensive feature importance analysis using SHAP (SHapley Additive exPlanations). In another study,^16^ integrated machine learning models and global sensitivity analysis to predict flexural strength in recycled aggregate-based concrete, showcasing how global sensitivity measures can highlight the influence of key input parameters in complex, sustainability-oriented mix designs. Similarly,^17^ presented ML-based predictions for concrete strength properties using coconut shell as partial coarse aggregate replacement, providing a more sustainable approach with minimal compromise on performance. Finally,^18^ investigated advanced models such as KNN, M5P, and Gradient Boosting to predict the flow of fly ash and blast furnace slag-based concrete, concluding that accurate flow prediction depends strongly on aggregate composition and water content.

Although these techniques provide significant insights, they often entail high computational expenses or rest on narrow assumptions, making practical adoption difficult. Deep learning, which has progressed considerably since the early backpropagation research^19^, displays promising capabilities in refining prediction accuracy and guiding complex optimization tasks. In particular, neural networks can manage diverse datasets and abstract important patterns to predict mechanical behavior more effectively than many traditional or shallow learning methods.

The current study proposes a unified framework wherein a deep learning model predicts the compressive strength of concrete mixes, while a multi-objective optimization routine seeks to maximize concrete strength, minimize overall costs, and reduce cement usage. This integrated setup harnesses neural networks for learning from existing data and employs multi-objective algorithms to handle conflicting targets methodically. Such an approach promotes data-driven decision-making that addresses both structural performance requirements and environmental sustainability. It also demonstrates practical value for industry stakeholders who need consistent and reproducible outcomes across differing material sources and field conditions.

The primary objectives of the study includes:

- Develop a deep learning-based predictive model for compressive strength using multiple concrete mix parameters.
- Integrate multi-objective optimization to minimize cost and cement usage while maintaining or improving strength.
- Validate the proposed framework under various design scenarios and highlight its potential for reducing carbon emissions.
- Provide insights and recommendations to researchers and industry practitioners for practical adoption in construction projects.

This paper is organized into clearly defined sections reflecting the logical flow of the research. Section "Exploratory data analysis" covers detailed dataset analysis and visualization of variables and their results. Section "Methodology" provides a comprehensive explanation of the methodology, including details on data handling, model formulation, and optimization procedures. Section "Results and discussion" describes the experimental outcomes and presents the optimized concrete mix designs, followed by a critical analysis of strengths and limitations. Section "Conclusion" offers conclusions drawn from the study and suggests directions for future research.

## Exploratory data analysis

The foundation of any robust machine learning model lies in the quality and comprehensiveness of the dataset utilized. In this study, the dataset was meticulously compiled from various experimental studies documented in the existing literature, resulting in a comprehensive single dataset suitable for analysis^20,21^. The dataset comprises a total of 1,133 samples, each characterized by nine numerical attributes: Cement, Blast Furnace Slag, Fly Ash, Water, Superplasticizer, Coarse Aggregate, Fine Aggregate, Age, and Compressive Strength. Among these, Compressive Strength serves as the output variable, while the remaining eight variables act as input features influencing the strength of concrete.

### Variable description and units

Understanding the variables involved is crucial for interpreting the results and ensuring the relevance of the analysis to practical applications. Table 1 provides a detailed description of each variable, including their respective units and a brief description of their role in the concrete mix design.

**Table 1:** Variable names, units, and descriptions.

**Variable**	**Unit**	**Description**
Cement	kg/m$^3$	Amount of cement in the mix
Blast Furnace Slag	kg/m$^3$	Quantity of blast furnace slag used as a supplementary cementitious material
Fly Ash	kg/m$^3$	Volume of fly ash added to enhance workability and reduce cement content
Water	kg/m$^3$	Water content in the concrete mix
Superplasticizer	kg/m$^3$	Amount of superplasticizer used to improve workability without increasing water content
Coarse Aggregate	kg/m$^3$	Quantity of coarse aggregates such as gravel or crushed stone
Fine Aggregate	kg/m$^3$	Amount of fine aggregates like sand
Age	days	Curing age of the concrete samples
Compressive Strength	MPa	Measured compressive strength of the concrete

### Descriptive analysis

The descriptive statistics presented in Table 2 offer valuable insights into the distribution and variability of each variable within the dataset. Furthermore, Table 2 presents a comprehensive descriptive analysis of the dataset, highlighting key statistical measures for each variable. These measures include the mean, standard deviation, minimum and maximum values, variance, skewness, kurtosis, first quartile (Q1), third quartile (Q3), and the interquartile range (IQR). For instance, the mean cement content is 276.50 kg/m$^3$ with a standard deviation of 103.47, indicating a significant variation in cement usage across different concrete mixes. The presence of a minimum value of 102 kg/m$^3$ and a maximum of 540 kg/m$^3$ further emphasizes the diversity in mix designs.

Similarly, Blast Furnace Slag and Fly Ash exhibit high variability, with means of 74.27 kg/m$^3$ and 62.81 kg/m$^3$ respectively, and standard deviations exceeding 70 kg/m$^3$. Notably, both these supplementary cementitious materials have minimum values of zero, suggesting that some mixes do not incorporate these components. This variability is crucial for understanding the potential impact of these materials on concrete properties.

The water content has a relatively narrow range, with a mean of 182.98 kg/m$^3$ and a standard deviation of 21.71 kg/m$^3$, indicating controlled water usage to maintain workability without compromising strength. Superplasticizer usage also varies significantly, with a mean of 6.42 kg/m$^3$ and a maximum value of 32.2 kg/m$^3$, highlighting its role in enhancing workability and strength.

Aggregate components show high consistency, particularly Coarse Aggregate and Fine Aggregate, with means of 964.83 kg/m$^3$ and 770.49 kg/m$^3$ respectively. The low skewness values for these aggregates suggest a symmetric distribution, which is favorable for model training and prediction.

Age exhibits extreme variability with a mean of 44.06 days and a standard deviation of 60.44 days, ranging from 1 to 365 days. This wide range captures the curing duration’s influence on compressive strength, a critical factor in concrete performance.

Finally, the Compressive Strength, the primary output variable, has a mean of 35.84 MPa and a standard deviation of 16.10 MPa, indicating substantial variability. The skewness and kurtosis values suggest a slight positive skew and platykurtic distribution, respectively, which may influence the choice of predictive modeling techniques.

**Table 2:** Descriptive statistics of the dataset.

${\textbf {Variable}}^{1}$	${\textbf {Mean}}^{2}$	${\textbf {Std. Dev.}}^{3}$	${\textbf {Min}}^{4}$	${\textbf {Median}}^{5}$	${\textbf {Max}}^{6}$	${\textbf {Var.}}^{7}$	${\textbf {Skew.}}^{8}$	${\textbf {Kurt.}}^{9}$	${\textbf {Q1}}^{10}$	${\textbf {Q3}}^{11}$	${\textbf {IQR}}^{12}$
Cement	276.50	103.47	102	266	540	10706.03	0.53	-0.46	190	342	152
Blast Furnace Slag	74.27	84.25	0	26	359.4	7097.52	0.77	-0.48	0	141.3	141.3
Fly Ash	62.81	71.58	0	0	260	5124.15	0.61	-0.91	0	121.97	121.97
Water	182.98	21.71	121.75	185.7	247	471.49	0.09	0.07	167	193.8	26.8
Superplasticizer	6.42	5.80	0	6.7	32.2	33.60	0.84	1.46	0	10.16	10.16
Coarse Aggregate	964.83	82.79	708	966.8	1145	6853.89	-0.17	-0.40	919	1026.6	107.6
Fine Aggregate	770.49	79.37	594	777.5	992.6	6300.21	-0.19	-0.17	720	821	101
Age	44.06	60.44	1	28	365	3653.15	3.47	13.81	14	28	14
Compressive Strength	35.84	16.10	2.33	34.67	82.60	259.23	0.42	-0.16	24.39	44.87	20.47

$^{1}$Variable: The parameter or characteristic being measured. $^{2}$Mean: The average value of the variable. $^{3}$Std. Dev.: Standard deviation, indicating the variability of the data. $^{4}$Min: Minimum value observed in the data. $^{5}$Median: The middle value when data is ordered. $^{6}$Max: Maximum value observed in the data. $^{7}$Var.: Variance, representing the spread of data points. $^{8}$Skew.: Skewness, indicating the asymmetry of the data distribution. $^{9}$Kurt.: Kurtosis, indicating the peakedness of the data distribution. $^{10}$Q1: First quartile, representing the 25th percentile of the data. $^{11}$Q3: Third quartile, representing the 75th percentile of the data. $^{12}$IQR: Interquartile range, the difference between Q3 and Q1.

### Data visualization

**Fig. 1:**
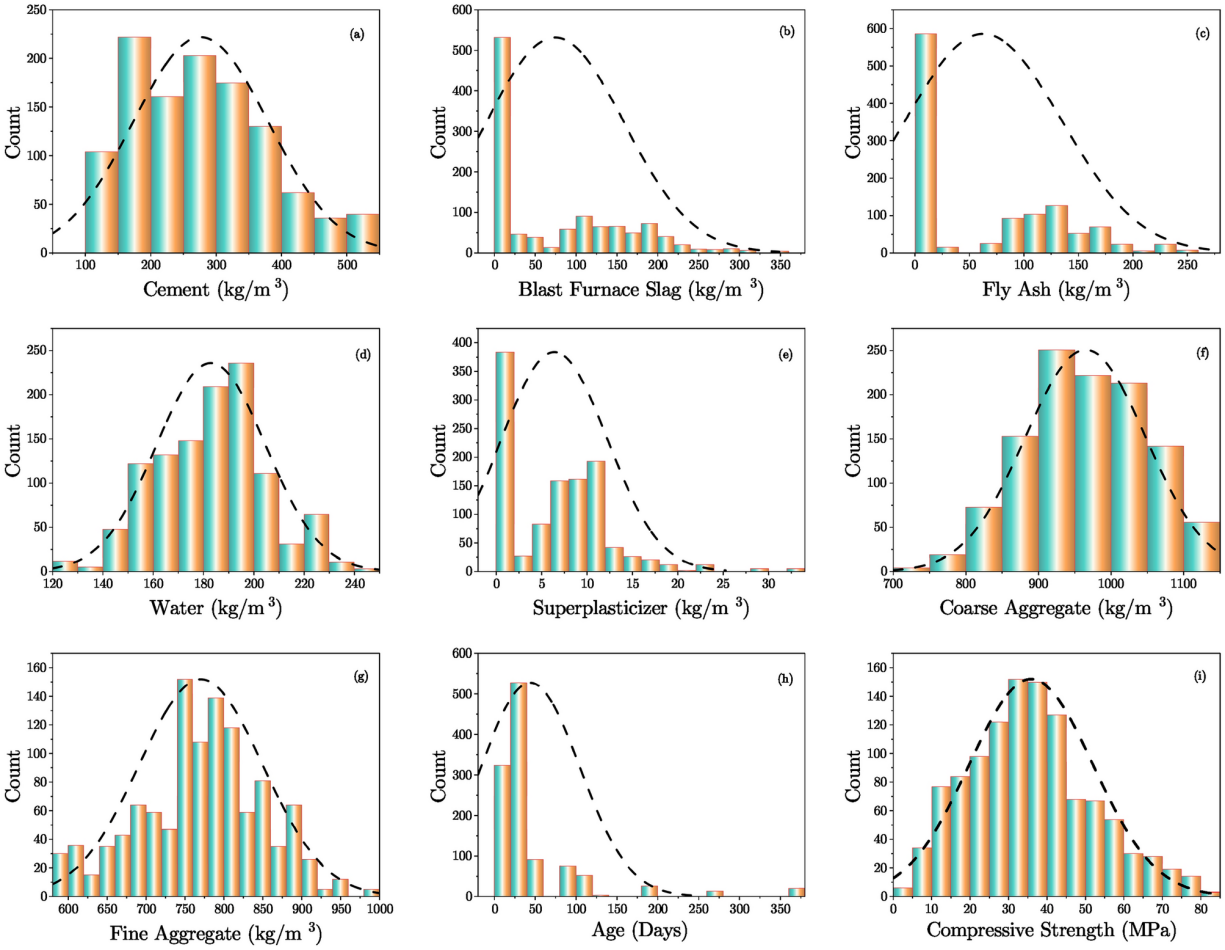
Histogram distributions with KDE plots for all variables.

**Fig. 2:**
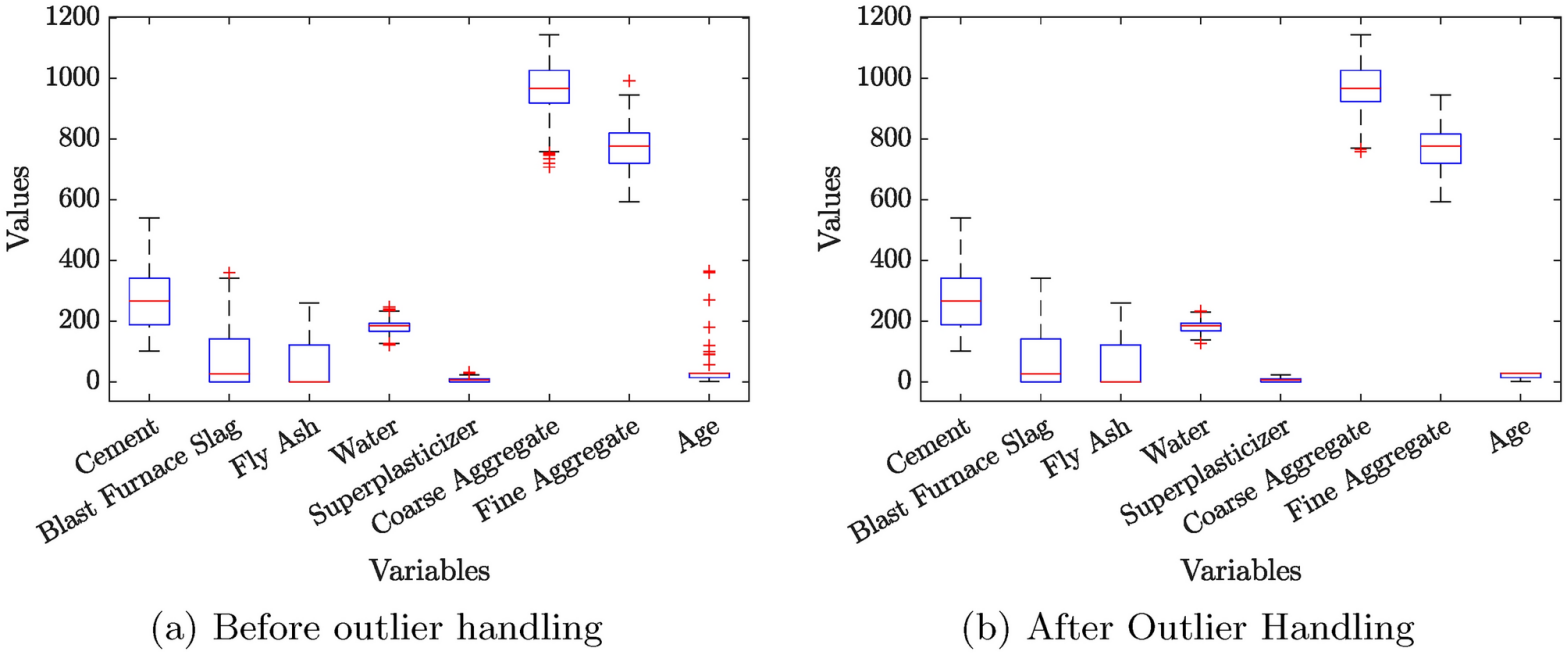
Boxplots before and after outlier handling for all variables.

**Fig. 3:**
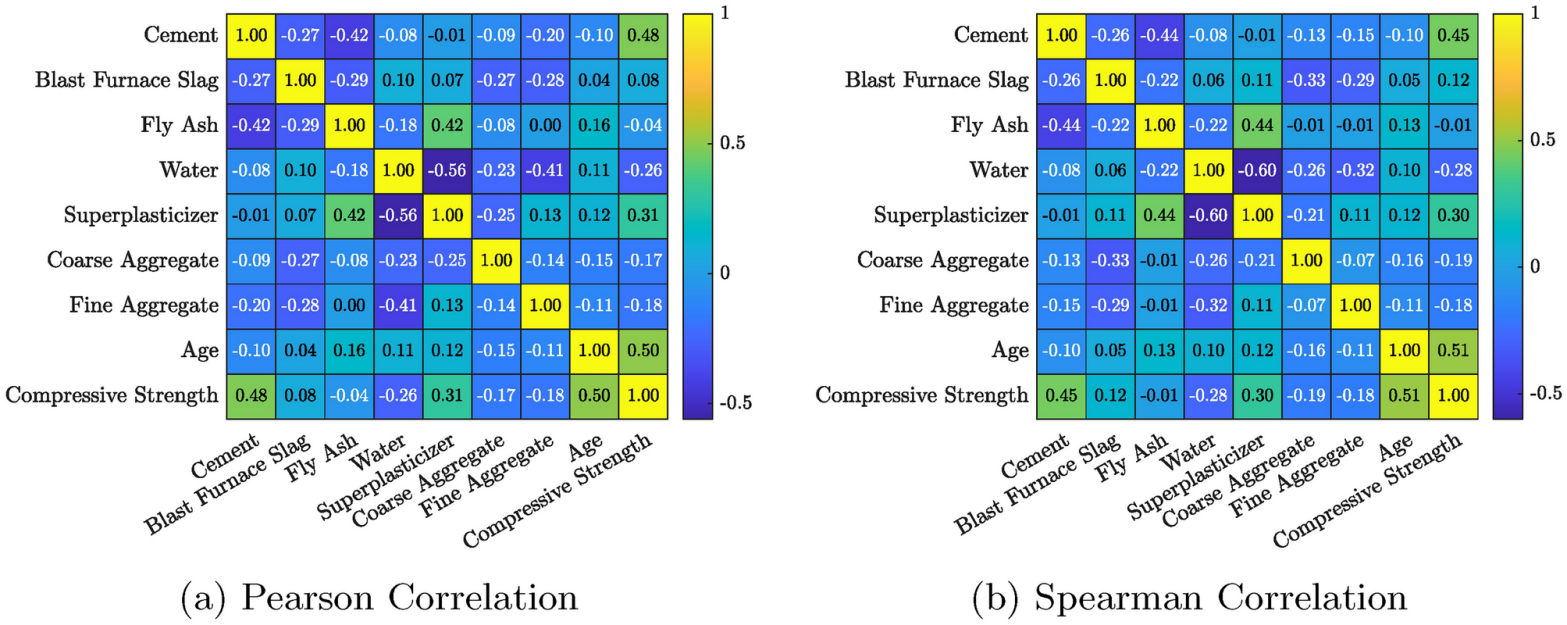
Correlation heatmaps of variables in dataset.

To gain a deeper understanding of the dataset’s distribution and relationships among variables, several visualization techniques were employed. Figures 1, 2, and 3 present the histogram distributions with Kernel Density Estimation (KDE) plots, box plots before and after outlier handling in the input variables of the dataset, and Pearson & Spearman correlation heatmaps, respectively.

Figure 1 provides a comprehensive visual analysis of the distribution of all variables in the dataset through histograms coupled with KDE plots. These visualizations reveal the central tendencies, variability, and potential anomalies in the data, complementing the detailed numerical insights in **Table** 2. For instance, the distribution of Cement (Fig. 1(a)) exhibits a near-symmetric bell-shaped curve, with the mean (276.50 $\hbox {kg/m}^{3}$) and median (266 $\hbox {kg/m}^{3}$) closely aligned, suggesting a relatively balanced usage across the dataset. The skewness (0.53) and kurtosis (-0.46) values further confirm the moderate deviation from symmetry.

Conversely, variables such as Blast Furnace Slag (Fig. 1(b)) and Fly Ash (Fig. 1(c)) display positively skewed distributions with significant concentrations around zero, as reflected by their high skewness values (0.77 and 0.61, respectively). These distributions imply limited usage of these components in many samples.

Water (**Fig.** 1(d)) shows a relatively narrow spread around its mean (182.98 $\hbox {kg/m}^{3}$) with low variability (standard deviation: 21.71), indicating consistent use. Similarly, Superplasticizer (Fig. 1(e)) highlights a sharp skewness (0.84) and higher kurtosis (1.46), indicative of a few samples with significantly higher values.

The distributions of Coarse Aggregate (Fig. 1(f)) and Fine Aggregate (Fig. 1(g)) are fairly symmetric with negative skewness values (-0.17 and -0.19, respectively), denoting a slight concentration towards higher values. Their interquartile ranges (107.6 $\hbox {kg/m}^{3}$ and 101 $\hbox {kg/m}^{3}$, respectively) also suggest consistent ranges of usage.

The Age variable (Fig. 1(h)) stands out with a highly skewed distribution (skewness: 3.47) and an extensive range from 1 to 365 days. Most samples concentrate around shorter curing periods, as evident from the median (28 days) and the interquartile range (14 days). This skewed nature reflects a dataset focused on early strength assessments.

Lastly, Compressive Strength (Fig. 1(i)) demonstrates a slight positive skewness (0.42), with most values centered around its mean (35.84 MPa) and median (34.67 MPa). The interquartile range (20.47 MPa) indicates significant variability, which is essential for analyzing strength development across diverse concrete mixes.

The combination of Fig. 1 and Table 2 provides a clear depiction of both the statistical and visual aspects of the dataset, highlighting key trends, variability, and potential data imbalances critical for subsequent analysis.

Box plots are highly effective in visualizing the presence of outliers, spread, and central tendencies of the data. Figure 2 illustrates the box plots for all variables in the dataset, comparing the distributions before and after outlier handling. These visualizations provide valuable insights into the data quality and the impact of outlier handling.

The initial box plots (Fig. 2a) reveal significant outliers in variables such as *Water*, *Superplasticizer*, *Coarse Aggregate*, and *Age*, which exhibit extreme values beyond their respective whiskers. For example, the *Superplasticizer* variable shows several high-value outliers, representing rare mix designs with a higher dosage. Similarly, *Age* demonstrates extreme values towards the upper end, which correspond to samples subjected to prolonged concrete maturity periods.

In contrast, variables like *Cement* and *Fly Ash* exhibit no observable outliers, as their distributions align well within the interquartile ranges. *Blast Furnace Slag* and *Fine Aggregate*, however, display a limited number of outliers, mostly concentrated towards higher values, reflecting their occasional high usage in specific concrete mixes.

The identified outlier are replaced with the median of that feature. Post outlier handling (Fig. 2b), the data distributions become more compact and consistent. The outliers in variables such as *Water*, *Superplasticizer*, *Coarse Aggregate*, and *Age* are addressed effectively, leading to a reduction in their variability and ensuring a more uniform distribution. For instance, the whiskers for *Superplasticizer* and *Age* now reflect a more realistic range, minimizing the influence of extreme values on subsequent analyses. The adjustments also enhance the representativeness of *Coarse Aggregate*, which initially exhibited significant variability.

Variables with minimal outliers initially, such as *Blast Furnace Slag* and *Fine Aggregate*, show only slight adjustments post-outlier handling, confirming their relative stability in the dataset. The consistent absence of outliers in *Cement* and *Fly Ash* remains unchanged, indicating their inherent uniformity across samples.

Overall, the comparison in Fig. 2 highlights the effectiveness of outlier handling in improving the dataset’s quality. By addressing extreme values in critical variables, the dataset becomes more balanced and better suited for predictive modeling, ensuring reliable and interpretable results.

Understanding the relationships between variables is pivotal for feature selection and model optimization. Figure 3 displays Pearson^22^ and Spearman correlation^23^ heatmaps, providing insights into both linear and monotonic relationships among the variables.

The Pearson correlation coefficient ($r$) quantifies the strength and direction of the linear relationship between two variables and is given by Eq. (1).

1 $$\begin{aligned} r = \frac{\sum _{i=1}^{n}(x_i - \bar{x})(y_i - \bar{y})}{\sqrt{\sum _{i=1}^{n}(x_i - \bar{x})^2 \sum _{i=1}^{n}(y_i - \bar{y})^2}}, \end{aligned}$$

where $x_i$ and $y_i$ are the individual sample points of the two variables, $\bar{x}$ and $\bar{y}$ are their respective means, and $n$ is the total number of observations. Pearson correlation captures the degree to which two variables change together linearly.

The Spearman rank correlation coefficient ($\rho$) evaluates the monotonic relationship between two variables, irrespective of whether it is linear. It is calculated using **Eq.** (2).

2 $$\begin{aligned} \rho = 1 - \frac{6 \sum _{i=1}^{n} d_i^2}{n(n^2 - 1)}, \end{aligned}$$

where $d_i$ is the difference between the ranks of corresponding values of $x$ and $y$, and $n$ is the number of observations. Unlike Pearson, Spearman correlation is robust to outliers and captures non-linear monotonic trends.

Both correlation coefficients provide complementary insights. While Pearson correlation emphasizes linear relationships, Spearman correlation detects rank-based monotonic trends. This dual analysis is crucial for identifying influential predictors. For example, the strong monotonic association between *Age* and *Compressive Strength* in Spearman analysis confirms its importance for predicting strength, even though its linear correlation is slightly weaker.

The Pearson correlation heatmap (Fig. 3a) reveals a moderately strong positive correlation between Cement and Compressive Strength ($r = 0.48$), indicating a direct linear relationship. The strongest negative correlation is observed between Fly Ash and Cement ($r = -0.42$), due to their partial substitution roles. These correlation values lie well within the range of $-1$ to $+1$, where values close to $|1|$ signify stronger linear relationships. The observed range of Pearson coefficients in this dataset spans from $-0.56$ (Water-Superplasticizer) to $+0.50$ (Age-Strength), suggesting varied yet meaningful dependencies across features. The Spearman heatmap (Fig. 3b) supports this finding with a similar monotonic relationship ($\rho = 0.45$). This consistency across both correlation measures highlights *Cement* as a key predictor for *Compressive Strength*.

For *Blast Furnace Slag*, a weak negative correlation with *Cement* is observed in Pearson ($r = -0.27$) and Spearman ($\rho = -0.26$), reflecting its role as a partial cement replacement. Similarly, *Fly Ash* demonstrates a stronger negative linear relationship with *Cement* ($r = -0.42$, $\rho = -0.44$), which aligns with its usage patterns in mix designs.

*Water* exhibits a weak negative correlation with *Compressive Strength* in both Pearson ($r = -0.26$) and Spearman ($\rho = -0.27$), confirming the detrimental effect of excessive water content on concrete strength. Furthermore, *Superplasticizer* shows a positive association with *Compressive Strength* ($r = 0.31$, $\rho = 0.30$), suggesting its beneficial role in optimizing water-cement ratios and improving concrete properties.

A notable monotonic relationship is observed between *Age* and *Compressive Strength*, with Pearson ($r = 0.50$) and Spearman ($\rho = 0.51$) indicating consistent strength gain over time. This result emphasizes the importance of age of concret for achieving desired concrete performance.

Inter-variable relationships also provide valuable insights. For example, *Water* exhibits a strong negative correlation with *Superplasticizer* ($r = -0.56$, $\rho = -0.60$), reflecting their inverse roles in maintaining workability. Similarly, *Coarse Aggregate* shows weak negative correlations with *Cement* and *Fly Ash*, which align with its role as a volume-filling component in concrete mix designs.

Overall, these correlation analyses highlight the importance of variables such as *Cement*, *Age*, and *Water* in predicting *Compressive Strength*. These findings guide the feature selection process by identifying influential variables and interdependencies, enabling more efficient and accurate predictive modeling.

## Methodology

**Fig. 4:**
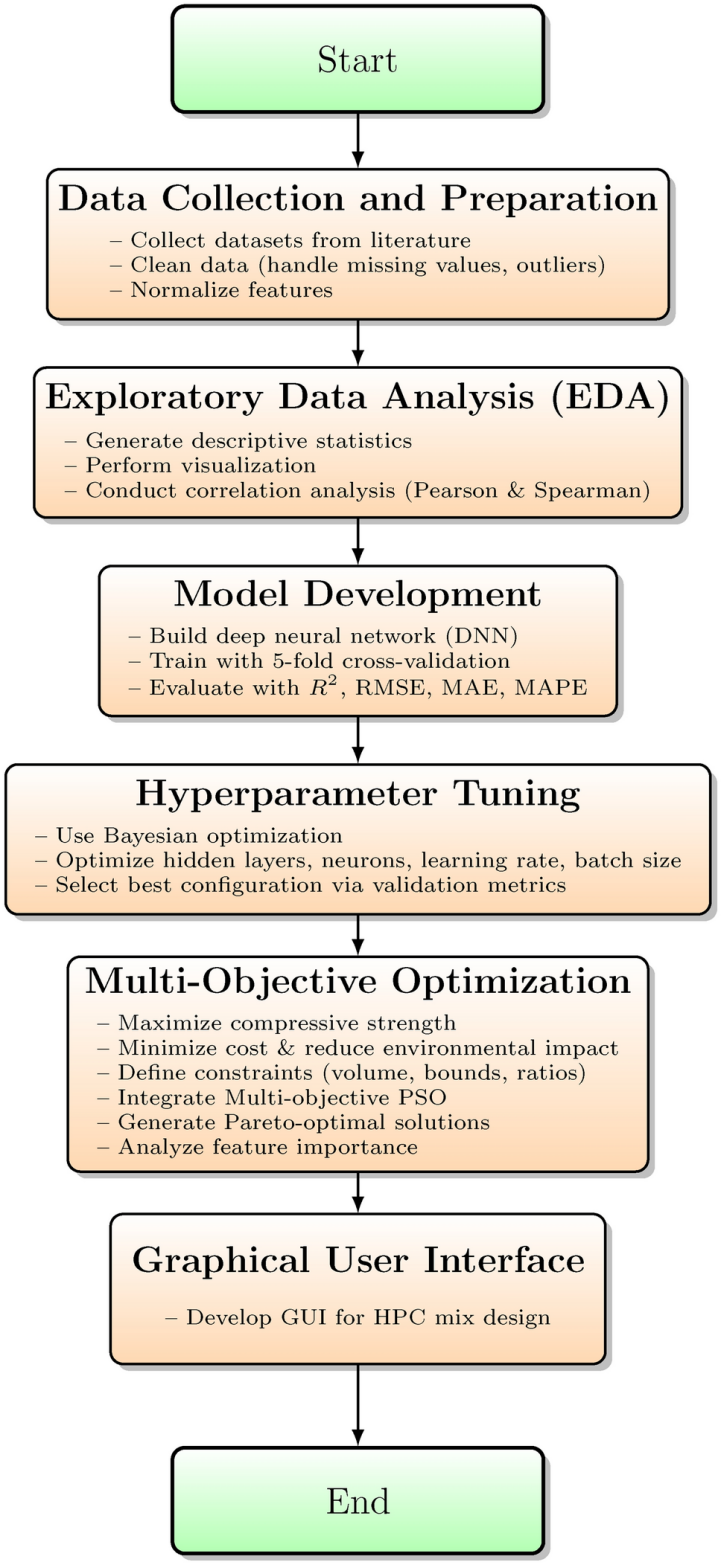
Flowchart of the proposed research methodology.

This section provides a comprehensive description of the systematic approach adopted for optimizing green concrete mix designs using deep learning and multi-objective optimization techniques. The methodology comprises data preprocessing, model development, hyperparameter optimization, model evaluation and validation, multi-objective optimization, feature importance analysis, and the development of a graphical user interface (GUI) to facilitate real-time mix design selection. The overall workflow is depicted in Fig. 4.

### Data preprocessing

Data preprocessing involves handling missing values, detecting and treating outliers, and feature normalization. Missing values are imputed using the median value of the respective feature. Outliers are detected using the interquartile range (IQR) method and replaced with the median value. In order to ensure that the learning algorithms converge efficiently during backward error propagation, the input features are scaled using min-max normalization. The normalization is performed using Eq. (3).

3 $$\begin{aligned} x_{\text {norm}} = \frac{x - \min (x)}{\max (x) - \min (x)}, \end{aligned}$$

where $x$ is the original feature value, and $\min (x)$ and $\max (x)$ denote the minimum and maximum values of the feature, respectively.

### Model development

**Fig. 5:**
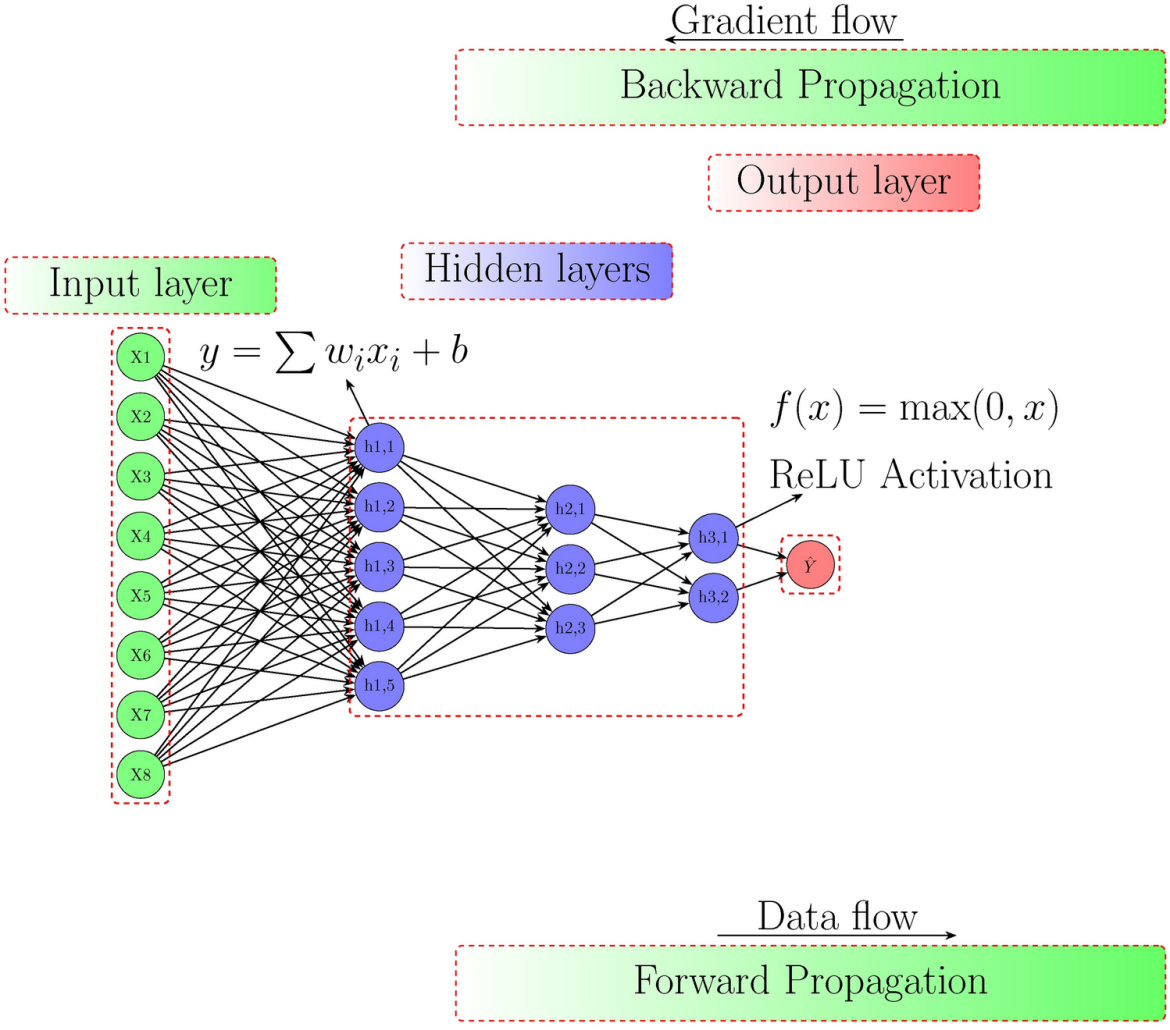
Architecture of the deep neural network model used for compressive strength prediction.

A deep neural network (DNN) is developed to model the complex relationships between the input variables and the compressive strength of concrete. The network comprises an input layer, multiple hidden layers with non-linear activation functions (ReLU), and an output layer that produces a scalar prediction of compressive strength. Mathematically, the forward propagation of a two-hidden-layer DNN can be described by using Eq. (4).

4 $$\begin{aligned} \textbf{h}_1&= \textrm{ReLU}(\textbf{W}_1 \textbf{x} + \textbf{b}_1), \quad \textbf{h}_2 = \textrm{ReLU}(\textbf{W}_2 \textbf{h}_1 + \textbf{b}_2) \nonumber \\ \hat{y}&= \textbf{W}_3 \textbf{h}_2 + b_3 \end{aligned}$$

where $\textbf{x}$ is the normalized input vector, $\textbf{W}_i$ and $\textbf{b}_i$ denote the weight matrices and bias vectors at layer $i$, respectively, and $\hat{y}$ is the predicted compressive strength. The detailed DNN model working architecture is depicted in **Fig.** 5 with layers, data and error propogation flow.

### Hyperparameter optimization

Hyperparameter tuning is a critical step in designing an effective DNN model. In this study, Bayesian optimization is employed to systematically search for the optimal hyperparameter configuration that minimizes the average Root Mean Squared Error (RMSE) computed over cross-validation folds.

Bayesian Optimization (BO) provides an efficient and probabilistically principled framework for optimizing objective functions, especially when model evaluations are computationally expensive, as in deep learning or complex ensemble models. Unlike traditional grid or random search strategies, BO intelligently selects the most promising hyperparameter configurations using acquisition functions and surrogate models, such as Gaussian Processes. This targeted exploration allows BO to converge more rapidly to optimal solutions, which is particularly advantageous in nonconvex optimization landscapes or when dealing with both continuous and discrete hyperparameters^24,25^.

In contrast, metaheuristic algorithms such as Genetic Algorithms (GA), Grey Wolf Optimization (GWO), and Differential Evolution (DE) have been extensively utilized in the modeling of concrete properties due to their robustness in handling nonlinear, multimodal optimization problems. These approaches, however, often require a significantly larger number of evaluations to achieve competitive performance^26–28^. For instance, in benchmark assessments comparing hybridized GWO- and DE-based models to BO-optimized alternatives, BO demonstrated faster convergence to higher performance with fewer function evaluations^29,30^.

Moreover, studies leveraging hybrid frameworks combining BO with machine learning models-such as XGBoost or deep neural networks-have reported enhanced predictive accuracy for high-performance and sustainable concrete formulations^24,30^. These findings affirm the suitability of BO in real-world engineering tasks where computational efficiency and performance precision are paramount. Bayesian optimization is a sequential design strategy for global optimization of expensive-to-evaluate functions. It is particularly well-suited for hyperparameter tuning because each evaluation (i.e., training the DNN) is computationally intensive. The key idea is to construct a probabilistic surrogate model for the objective function, typically using a Gaussian Process (GP)^31^, and to use an acquisition function to guide the search for the next hyperparameter set.

Let $f(\theta )$ denote the objective function (in our case, the average RMSE over cross-validation) as a function of the hyperparameters $\theta$. Bayesian optimization builds a surrogate model $\hat{f}(\theta )$ that approximates $f(\theta )$. A common choice for the surrogate is a Gaussian Process, which assumes that the objective function follows a multivariate Gaussian distribution. The GP is characterized by its mean function $\mu (\theta )$ and covariance function $k(\theta , \theta ')$. The prediction at an unseen hyperparameter $\theta _*$ is given by Eq. (5).

5 $$\begin{aligned} \hat{f}(\theta _*) = \mu (\theta _*) + k(\theta _*, \Theta ) k(\Theta ,\Theta )^{-1} (f(\Theta ) - \mu (\Theta )), \end{aligned}$$

where $\Theta$ represents the set of hyperparameters already evaluated, and $f(\Theta )$ is the vector of corresponding objective values.

The acquisition function, $a(\theta )$, balances exploration (sampling hyperparameters in regions of high uncertainty) and exploitation (sampling where the surrogate model predicts low RMSE). One popular choice is the Expected Improvement (EI) function, which is given by Eq. (6).

6 $$\begin{aligned} \text {EI}(\theta ) = \mathbb {E}\left[ \max \left( f_{\min } - \hat{f}(\theta ), 0\right) \right] , \end{aligned}$$

where $f_{\min }$ is the best (i.e., minimum) observed RMSE so far. The next hyperparameter configuration to evaluate is chosen by maximizing the acquisition function, calculated using Eq. (7).

7 $$\begin{aligned} \theta _{\text {next}} = \arg \max _{\theta } \; \text {EI}(\theta ). \end{aligned}$$

This iterative process continues until a predefined stopping criterion (maximum number of evaluations or a convergence threshold) is reached.

The rationale for selecting specific hyperparameters for the DNN is based on prior knowledge of the problem and empirical studies. In our case, the hyperparameters tuned include:

- Number of hidden units: Determines the capacity of the network to learn complex nonlinear relationships. Too few units may lead to underfitting, while too many can cause overfitting and increased computational cost.
- Learning rate: Controls the step size during gradient descent. A smaller learning rate may result in slow convergence, whereas a larger learning rate could lead to divergence.
- Mini-batch size: Influences the stability of the weight updates and the convergence rate during training.

Table 3 lists the hyperparameters and their corresponding search ranges used during Bayesian optimization.

**Table 3:** DNN hyperparameters and search ranges for Bayesian optimization.

Hyperparameter	Type	Range
Number of Hidden Units 1	Integer	8 to 32
Number of Hidden Units 2	Integer	4 to 16
Learning Rate	Continuous (log-scale)	$10^{-4}$ to $10^{-2}$
Mini-batch Size	Integer	16 to 64

The following algorithm (see Algorithm 1) outlines the complete steps for optimizing the DNN hyperparameters using Bayesian optimization.

**Algorithm 1:**
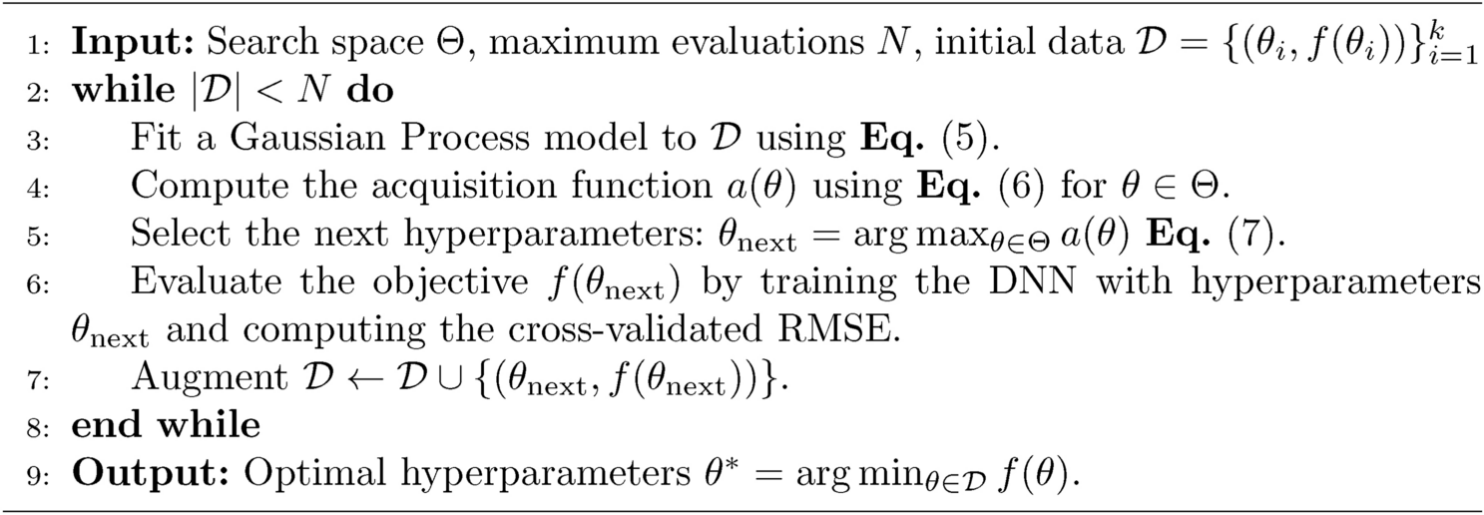
Bayesian optimization for DNN hyperparameter tuning.

Bayesian optimization, by modeling the objective function probabilistically and iteratively updating the surrogate model using Eq. (5), allows us to efficiently navigate the high-dimensional hyperparameter space. The use of the EI acquisition function defined in Eq. (6) and the subsequent selection via Eq. (7) ensures that the hyperparameters chosen for the final DNN configuration lead to the best generalization performance on unseen data.

### Model evaluation and validation

The performance of the developed DNN model is evaluated on unseen test data using several complementary metrics. These metrics are chosen to capture different aspects of model prediction accuracy and reliability. In particular, the following metrics are considered:

- **Root Mean Squared Error (RMSE):**
  8 $$\begin{aligned} \textrm{RMSE} = \sqrt{\frac{1}{N} \sum _{i=1}^{N} \left( \hat{y}_i - y_i\right) ^2}, \end{aligned}$$
  where $N$ is the number of observations, $\hat{y}_i$ is the predicted value, and $y_i$ is the true value. RMSE is highly sensitive to large errors due to the squaring operation and provides an overall measure of prediction variance. This metric is particularly useful when large deviations are undesirable, as described in Eq. (8).
- **Mean Absolute Error (MAE):**
  9 $$\begin{aligned} \textrm{MAE} = \frac{1}{N} \sum _{i=1}^{N} \left| \hat{y}_i - y_i\right| , \end{aligned}$$
  MAE computes the average magnitude of errors without considering their direction. Unlike RMSE, it does not disproportionately penalize large errors, which makes it a straightforward and interpretable measure of error magnitude as shown in Eq. (9).
- **Mean Absolute Percentage Error (MAPE):**
  10 $$\begin{aligned} \textrm{MAPE} = \frac{100}{N} \sum _{i=1}^{N} \left| \frac{\hat{y}_i - y_i}{y_i}\right| , \end{aligned}$$
  MAPE expresses the average error as a percentage, facilitating comparisons across different scales and contexts. A lower MAPE indicates that the model’s predictions are, on average, closer to the actual values in relative terms, as illustrated in Eq. (10).
- **Coefficient of determination** ($R^2$):
  11 $$\begin{aligned} R^2 = 1 - \frac{\sum _{i=1}^{N}\left( \hat{y}_i - y_i\right) ^2}{\sum _{i=1}^{N}\left( y_i - \bar{y}\right) ^2}. \end{aligned}$$
  $R^2$ quantifies the proportion of variance in the observed data that is explained by the model. A higher $R^2$ (closer to 1) indicates that the model captures a significant portion of the variance in the data, as given in Eq. (11).

Each metric serves a specific purpose: a low RMSE and MAE indicate that the model predictions are numerically close to the true values; a low MAPE suggests that the relative error is small; and a high $R^2$ signifies that the model is effective in explaining the variability in the data.

To provide a single unified measure that accounts for all these individual metrics, a composite metric is introduced. This metric aggregates the normalized individual metrics using a weighted sum:

12 $$\begin{aligned} M_{\text {composite}} = w_1 \frac{\textrm{RMSE}}{\textrm{RMSE}_{\text {ref}}} + w_2 \frac{\textrm{MAE}}{\textrm{MAE}_{\text {ref}}} + w_3 \frac{\textrm{MAPE}}{\textrm{MAPE}_{\text {ref}}} + w_4 \left( 1 - R^2\right) , \end{aligned}$$

where $w_1, w_2, w_3,$ and $w_4$ are the weights assigned to RMSE, MAE, MAPE, and $R^2$, respectively, with $w_1+w_2+w_3+w_4 = 1$, and $\textrm{RMSE}_{\text {ref}}$, $\textrm{MAE}_{\text {ref}}$, and $\textrm{MAPE}_{\text {ref}}$ are reference values used for normalization (for example, the maximum observed values for each metric). Equation (12) provides an overall performance score that simplifies the comparison among different models or hyperparameter settings.

The rationale behind selecting these evaluation metrics is that they collectively provide a multi-faceted view of model performance. RMSE emphasizes the impact of large errors, MAE gives a clear average error magnitude, and MAPE normalizes errors as percentages to facilitate comparisons across different scales. The $R^2$ metric further elucidates the model’s ability to capture the variance in the data. By combining these metrics into a composite measure via a weighted technique, one obtains a robust indicator that can be used to compare model performance comprehensively. A well-performing model is expected to exhibit low RMSE, MAE, and MAPE values along with a high $R^2$, resulting in a low composite metric value.

In summary, the individual metrics are employed to evaluate different dimensions of the DNN’s performance, while the composite metric unifies these measures into a single score that reflects the overall predictive capability of the model.

### Multi-objective optimization

To design an optimal concrete mix, a multi-objective optimization framework is formulated that simultaneously addresses three conflicting objectives: (i) maximizing compressive strength, (ii) minimizing cost, and (iii) minimizing cement consumption to reduce the environmental impact. In this formulation, the compressive strength is predicted by the DNN model, the cost is computed as the weighted sum of the masses of the ingredients (using their respective per unit costs), and the third objective explicitly minimizes cement content.

#### Objective functions

The first objective is to maximize the compressive strength predicted by the DNN. Since optimization algorithms are conventionally formulated as minimization problems, we define the strength objective as the negative of the predicted compressive strength:

13 $$\begin{aligned} f_1 = -\hat{y}, \end{aligned}$$

where $\hat{y}$ is the compressive strength predicted by the DNN model. This formulation (see Eq. (13)) ensures that lower values of $f_1$ correspond to higher compressive strength.

The second objective minimizes the cost of the concrete mix, which is computed as the sum of the products of the masses of the ingredients and their respective unit costs:

14 $$\begin{aligned} f_2 = \sum _{i=1}^{7} c_i x_i, \end{aligned}$$

where $x_i$ denotes the mass of the $i^{th}$ ingredient and $c_i$ is its unit cost in Indian rupees. Table 4 summarizes the unit average costs of each material used in the mix (see Eq. (14)). This objective ensures economic feasibility by selecting the mix proportions that result in the lowest overall cost.

**Table 4:** Unit average costs of concrete ingredients in indian rupees.

Ingredient	Unit Cost (Rs. per kg)
Cement	7
Blast Furnace Slag	10
Fly Ash	3
Water	1
Superplasticizer	130
Coarse Aggregate	0.8
Fine Aggregate	2.5

The third objective aims to reduce the environmental impact by minimizing the cement content:

15 $$\begin{aligned} f_3 = x_1, \end{aligned}$$

with $x_1$ representing the mass of cement (see Eq. (15)). Reducing cement consumption contributes directly to lowering the carbon footprint of the concrete.

Thus, the overall tri-objective optimization problem is formulated as:

$$\begin{aligned} \min _{x} \quad \{ f_1(x), f_2(x), f_3(x) \} \end{aligned}$$

with the understanding that a lower $f_1$ indicates higher strength, while lower $f_2$ and $f_3$ represent reduced cost and cement usage, respectively.

#### Constraints

In order to ensure that the generated mix designs are practical and compliant, several constraints are imposed while optim mix design of concrete:

1. **Volume constraint:** One cubic meter of concrete must be produced. This is represented by an equality constraint on the total mass of the ingredients:
  16 $$\begin{aligned} \sum _{i=1}^{7} x_i = M_{\text {total}}, \end{aligned}$$
  where $M_{\text {total}}$ is the total mass (in kg) required for 1 m$^3$ of concrete (typically around 2400–2500 kg), as shown in Eq. (16).
2. **Ratio constraint:** The water-cement ratio is constrained to a maximum value to ensure durability. The considered ratio constraint in the study is:
  17 $$\begin{aligned} \frac{x_4}{x_1} \le 0.6, \end{aligned}$$
  where $x_4$ is the mass of water and $x_1$ is the mass of cement (see Eq. (17)).
3. **Range constraints:** Each ingredient is restricted to lie within a predefined range based on experimental data:
  18 $$\begin{aligned} x_i^{\text {min}} \le x_i \le x_i^{\text {max}}, \quad i = 1,2,\ldots ,7, \end{aligned}$$
  as shown in Eq. (18). Table 5 provides the lower and upper bounds for each of the eight variables (including Age). The optimization bounds in Table 5 were calibrated to be both practical feasible and regulatory compliant, whereas the descriptive statistics in Table 2 cover the entire range of mix compositions in the Yeh dataset. For example, values of Cement exceeding 540 $\hbox {kg/m}^{3}$ or falling below 102 $\hbox {kg/m}^{3}$ in the original dataset were omitted because such values are not practical in most structural applications. An alternative approach, instead, was to provide bounds for critical constituents, cement, supplementary cementitious materials (SCMs), aggregates, and water, in accordance with the triad of Indian Standard IS 10262:2019 mix proportioning specifications, and empirical bounds based on the state-of-the-art high performance concrete research. Yet, empirical research including that by Gedam^32^ shows that these input values are fundamental in developing shrinks and creeps predictive modeling of highperformance concrete, and even empirical data can be used to set thresholds on the input parameters for prediction. The insights reinforce the need to exclude statistically valid but structurally unrealistic data points during optimization. Therefore, the bounds selected in Table 5 are a compromise, which maintains statistical representativeness and satisfies concrete mix design codes and sustainability constraints. Therefore, the Yeh dataset remains a necessary tool in constructing an informed statistical profile of mix parameters, and the refined bounds allow model outputs that are implementable and domain appropriate.

If a candidate solution violates any of these constraints, penalty factors are applied to its objective values to discourage infeasible designs.

The variable bounds for optimization were selected based on statistical analysis of the dataset and practical design guidelines for high-performance concrete. All aggregate values are assumed to be at their Saturated Surface Dry (SSD) condition, ensuring consistency with standard mix design practice. Additionally, in response to reviewer feedback, the optimization constraints now include not only individual bounds for cementitious materials (cement, slag, fly ash) but also an aggregate bound on the total blended binder content. The total cementitious content (i.e., cement + blast furnace slag + fly ash) in optimized mixes ranges between 310 kg/m$^3$ and 540 kg/m$^3$, aligning with industry practices and the range observed in the Yeh dataset. Table 5 presents the complete variable ranges used in the optimization. The total cementitious content (cement + slag + fly ash) is constrained to 310-540 kg/m$^3$.

**Table 5:** Lower and upper bounds for concrete Mix Ingredients (all aggregates in SSD condition).

**Variable**	**Lower Bound**	**Upper Bound**	**Unit**
Cement	310	450	kg/m$^3$
Blast Furnace Slag	0	200	kg/m$^3$
Fly Ash	0	135	kg/m$^3$
Water	125	250	kg/m$^3$
Superplasticizer	0	15	kg/m$^3$
Coarse Aggregate	750	1500	kg/m$^3$
Fine Aggregate	550	1250	kg/m$^3$
Age	1	365	days

#### Overall optimization formulation

The complete multi-objective optimization problem is thus formulated as:

$$\begin{aligned} \begin{aligned} \min _{x} \quad&\{ f_1(x), \, f_2(x), \, f_3(x) \} \\ \text {subject to} \quad&\sum _{i=1}^{7} x_i = M_{\text {total}}, \quad {\textbf {Eq.}}~(16) \\&\frac{x_4}{x_1} \le 0.6, \quad {\textbf {Eq.}}~(17) \\&x_i^{\text {min}} \le x_i \le x_i^{\text {max}}, \quad i = 1,\ldots ,7. \quad {\textbf {Eq.}}~(18) \end{aligned} \end{aligned}$$

This formulation enables the optimizer to identify a set of Pareto-optimal solutions that offer trade-offs among maximizing strength, minimizing cost, and reducing cement consumption.

#### Multi-objective particle swarm optimization

Particle Swarm Optimization (PSO) is a population-based stochastic optimization algorithm inspired by the social behavior of birds flocking or fish schooling. In the conventional PSO approach, each particle in the swarm updates its velocity and position based on its personal best position as well as the global best position found by the swarm. The velocity of each particle is updated using Eq. (19) whereas postion of is modified using Eq. (20) using obtained updated velocity of each particle.

19 $$\begin{aligned} & v_{i}^{t+1} = \omega v_{i}^{t} + c_1 r_1 (p_{i}^{t} - x_{i}^{t}) + c_2 r_2 (g^{t} - x_{i}^{t}) \end{aligned}$$

20 $$\begin{aligned} & x_{i}^{t+1} = x_{i}^{t} + v_{i}^{t+1} \end{aligned}$$

where:

- $v_{i}^{t}$ is the velocity of particle $i$ at iteration $t$,
- $x_{i}^{t}$ is the position of particle $i$ at iteration $t$,
- $p_{i}^{t}$ is the personal best position of particle $i$,
- $g^{t}$ is the global best position found so far,
- $\omega$ is the inertia weight,
- $c_1$ and $c_2$ are acceleration coefficients,
- $r_1$ and $r_2$ are random numbers uniformly distributed in $[0,1]$.

For multi-objective problems, PSO is extended to Multi-Objective PSO (MOPSO) by maintaining an archive of non-dominated solutions that collectively form the Pareto front.

In MOPSO, each particle’s velocity and position are updated as in conventional PSO (see Eqs. (19) and (20)). However, instead of maintaining a single global best, the algorithm maintains an archive of non-dominated solutions. During each iteration, particles update their personal bests based on Pareto dominance, and the archive is updated to reflect the current Pareto front. A leader is then selected from the archive (often randomly) to guide the swarm’s movement.

**Fig. 6:**
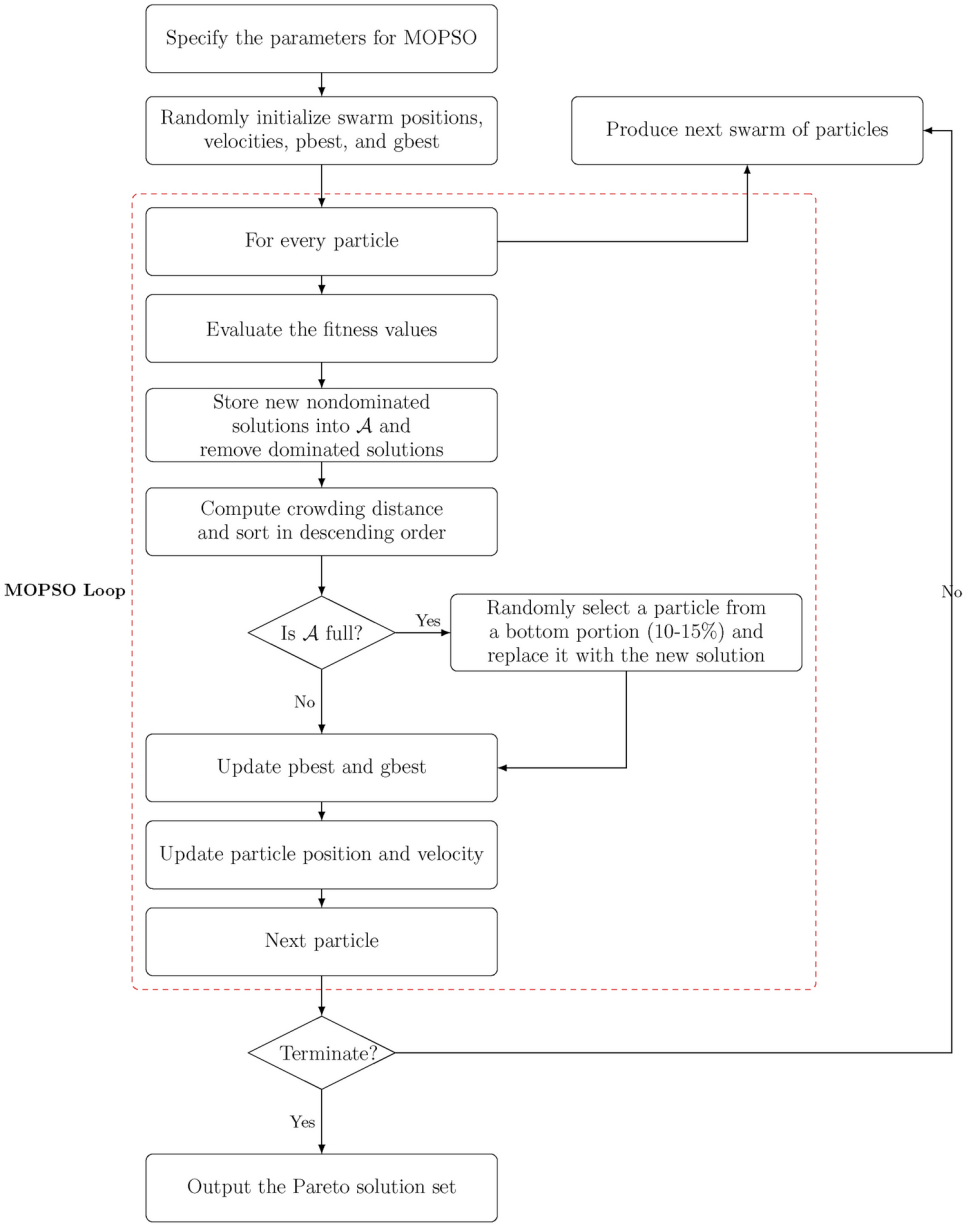
Flowchart of the MOPSO algorithm.

Figure 6 provides a flowchart summarizing the MOPSO algorithm, and Algorithm 2 presents the pseudocode for the complete procedure. Through this process, MOPSO effectively balances the conflicting objectives by exploring a diverse set of solutions and identifying a Pareto front that represents the trade-offs among maximizing strength, minimizing cost, and reducing cement consumption.

**Algorithm 2:**
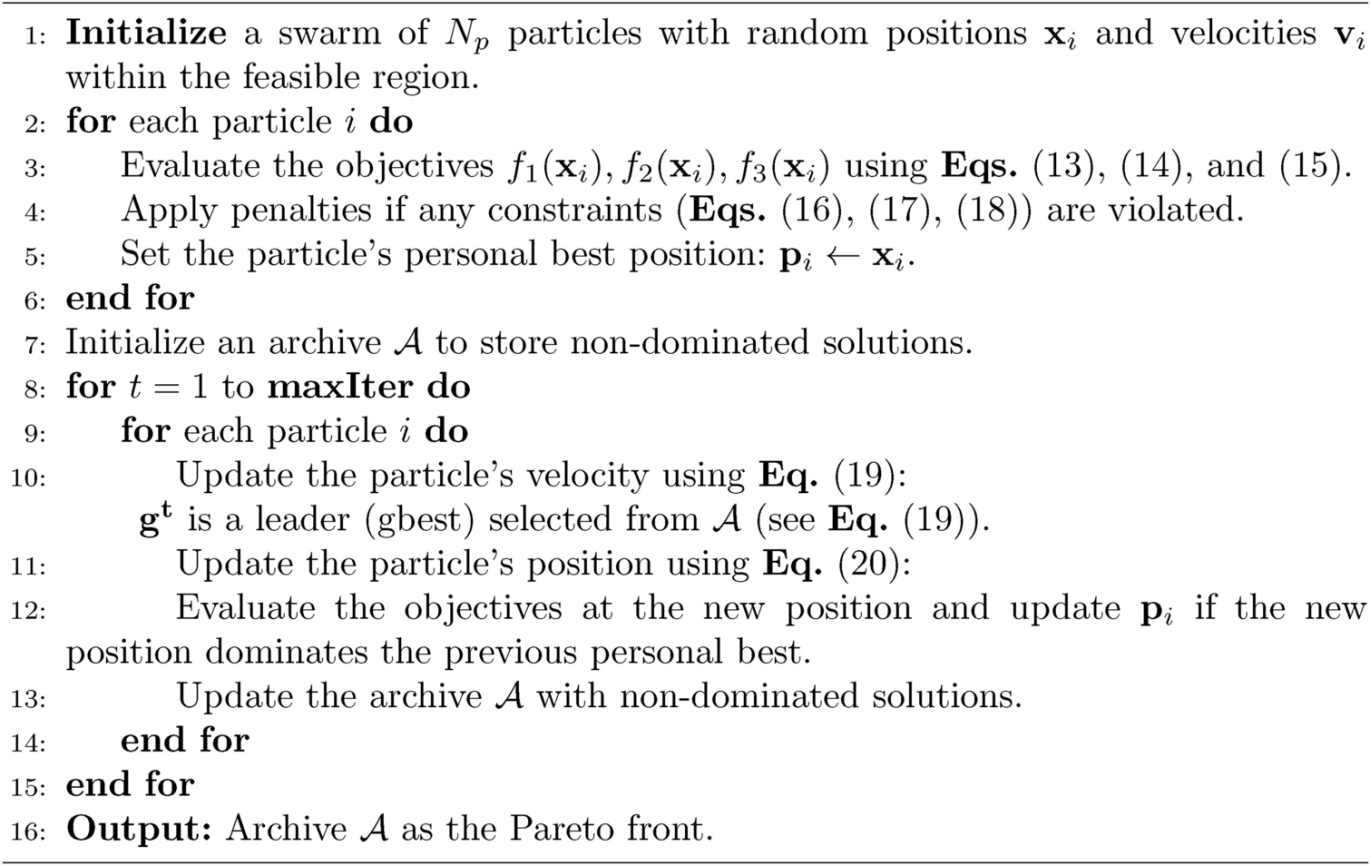
Multi-Objective Particle Swarm Optimization (MOPSO).

Although MOPSO is known to occasionally suffer from limited Pareto diversity or premature convergence, we mitigated these issues by integrating a crowding distance mechanism in the archive, stochastic leader selection, and adaptive reinitialization. Compared to algorithms like NSGA-II or MOEA/D, MOPSO was found to be better suited for this problem due to its lower computational overhead, which is essential for real-time integration within the GUI^33–35^. Future work may investigate hybridized MOPSO variants or adaptive $\varepsilon$-NSGA-III to further improve search efficiency.

### Feature importance analysis

To gain insights into the influence of individual input features on the predicted compressive strength, a permutation-based feature importance analysis is performed. This analysis is significant for two main reasons. First, it enhances the interpretability of the DNN model by revealing which input variables have the greatest impact on the prediction. Second, it provides practical guidance for material selection and mix design adjustments by identifying key factors that affect concrete strength.

In this study, the feature importance analysis is conducted by first computing a baseline performance metric on the original test dataset. Specifically, the baseline RMSE is calculated as follows:

21 $$\begin{aligned} \textrm{RMSE}_{\text {baseline}} = \sqrt{\frac{1}{N} \sum _{i=1}^{N} \left( y_i - \hat{y}_i\right) ^2}, \end{aligned}$$

where $y_i$ and $\hat{y}_i$ denote the actual and predicted compressive strengths, respectively, and $N$ is the total number of test samples (see Eq. (21)).

Next, for each feature $j$, its values in the test dataset are randomly permuted while all other features remain fixed. This permutation disrupts the relationship between the feature and the target variable, leading to a degradation in model performance if the feature is important. The RMSE is recalculated on the permuted test set:

22 $$\begin{aligned} \textrm{RMSE}_{\text {perm}}^{(j)} = \sqrt{\frac{1}{N} \sum _{i=1}^{N} \left( y_i - \hat{y}_i^{(j)}\right) ^2}, \end{aligned}$$

where $\hat{y}_i^{(j)}$ represents the prediction for sample $i$ after permuting the $j^{th}$ feature (see Eq. (22)). The importance of feature $j$ is then quantified by the increase in error:

23 $$\begin{aligned} I_j = \textrm{RMSE}_{\text {perm}}^{(j)} - \textrm{RMSE}_{\text {baseline}}. \end{aligned}$$

A larger value of $I_j$ indicates that permuting the feature has a significant adverse effect on the model’s performance, thereby marking it as an important predictor (see Eq. (23)).

The significance of this approach lies in its ability to provide a direct measure of how much each feature contributes to the predictive performance. By comparing the baseline RMSE with the RMSE after permutation, and then computing the difference, the analysis identifies which features the model relies on most for accurate predictions. This information is invaluable for both model validation and for practical decisions in concrete mix design.

### Graphical user interface

To facilitate practical deployment and enhance user accessibility, a comprehensive GUI is developed using MATLAB’s uifigure framework. The GUI is designed to allow users to easily generate eco-efficient concrete mix designs without requiring in-depth technical knowledge of the underlying optimization algorithms.

The interface is organized into two distinct modes. In the Ingredient Input Mode, users can manually input custom values for each of the seven concrete ingredients and the concrete age. These input fields are clearly labeled with units (kg/m$^3$ for material quantities and days for age) to ensure clarity and readability. Default values, derived from experimental data, are pre-populated to assist users in making informed modifications. This mode is particularly useful when users have specific material properties or site conditions that they wish to incorporate into the mix design.

In contrast, the Concrete Grade Mode offers a streamlined approach where users simply select a desired concrete grade from a comprehensive list ranging from M5 to M150. For grades at or below M20, the GUI provides a nominal mix design in accordance with IS guidelines. For grades above M20, the system employs a hybrid approach: it integrates the DNN-based model with a MOPSO algorithm. In this mode, the DNN predicts compressive strength based on the normalized input features, while MOPSO searches for optimal mix proportions under constraints such as the water–cement ratio (see Eq. (17)) and ingredient range limits (see Eq. (18)). The optimized solution provides the recommended proportions, the predicted compressive strength (via Eq. (13)), and the corresponding cost calculated using unit cost data (see Table 4 and Eq. (14)).

The design emphasizes user-friendliness by dynamically displaying the appropriate input controls based on the selected mode. When users switch to Concrete Grade Mode, the input fields corresponding to individual ingredients are hidden to avoid clutter and potential confusion, ensuring that only relevant controls (grade selection and age input) are displayed.

This GUI not only simplifies the interaction with complex optimization algorithms but also allows decision-makers to quickly evaluate mix designs based on key performance indicators such as compressive strength, cost, and cement consumption. By integrating advanced computational techniques with an intuitive user interface, the proposed framework bridges the gap between research and practical application in the field of eco-efficient concrete mix design.

## Results and discussion

### DNN model performance evaluation

The performance of the developed DNN model was assessed using 5-fold cross validation on the test set. The evaluation metrics computed for each fold include the $R^2$, RMSE, MAE, MAPE, and a composite metric $M_{\text {composite}}$ (see Eq. (12)). The results presented in Table 6 demonstrate the robust performance of the developed DNN model in predicting concrete compressive strength across 5-fold cross validation. The average coefficient of determination (cv$R^2$) of 0.9360 indicates that the model is able to explain approximately 93.6% of the variance in the experimental compressive strength values, reflecting a high level of predictive accuracy. This high $R^2$ value underscores the model’s ability to capture the underlying relationships between the mix design variables and the resulting strength.

Furthermore, the average Root Mean Squared Error (cvRMSE) is 5.7141 MPa and the Mean Absolute Error (cvMAE) is 4.3543 MPa, which suggest that the absolute prediction errors are relatively small in magnitude. These error metrics provide an insight into the variance and average deviation of the predictions from the true values. In addition, the Mean Absolute Percentage Error (cvMAPE) of 14.0472% indicates that, on average, the predictions deviate from the actual compressive strength by only 14% in relative terms. Such a level of relative error is acceptable given the inherent variability in concrete properties.

To further encapsulate the overall performance of the model, a composite metric $M_{\text {composite}}$ was introduced. With an average composite metric value of 0.7197 and low standard deviation across folds, this unified measure confirms the consistent and robust performance of the DNN model. The composite metric integrates the strengths of individual performance measures by assigning appropriate weights, thereby providing a single comprehensive indicator of model accuracy.

Overall, the low error metrics coupled with a high $R^2$ value imply that the DNN model exhibits excellent generalization capability. The consistency of the results across the 5-fold cross validation not only validates the model’s predictive accuracy but also instills confidence in its practical applicability for concrete mix design optimization. These findings have significant implications: the model’s reliability in predicting compressive strength forms a critical component in the subsequent multi-objective optimization process, ensuring that the generated mix designs meet both performance and sustainability objectives.

**Table 6:** DNN model performance across 5-fold cross-validation on the test set.

**Fold**	$\mathbf {cvR^2}$	$\textbf{cvRMSE}$ (MPa)	$\textbf{cvMAE}$ (MPa)	$\textbf{cvMAPE}$ (%)	$M_{\text {composite}}$
1	0.9531	5.7397	4.4737	15.1343	0.7410
2	0.8994	6.2257	4.3938	14.9978	0.7673
3	0.9155	5.7269	4.4951	14.9060	0.7473
4	0.9217	5.4772	4.2659	12.8882	0.6897
5	0.9900	5.4011	4.1432	12.3095	0.6532
**Average**	0.9360	5.7141	4.3543	14.0472	0.7197
**Std. Dev.**	0.0322	0.2886	0.1327	1.1988	0.0420

**Fig. 7:**
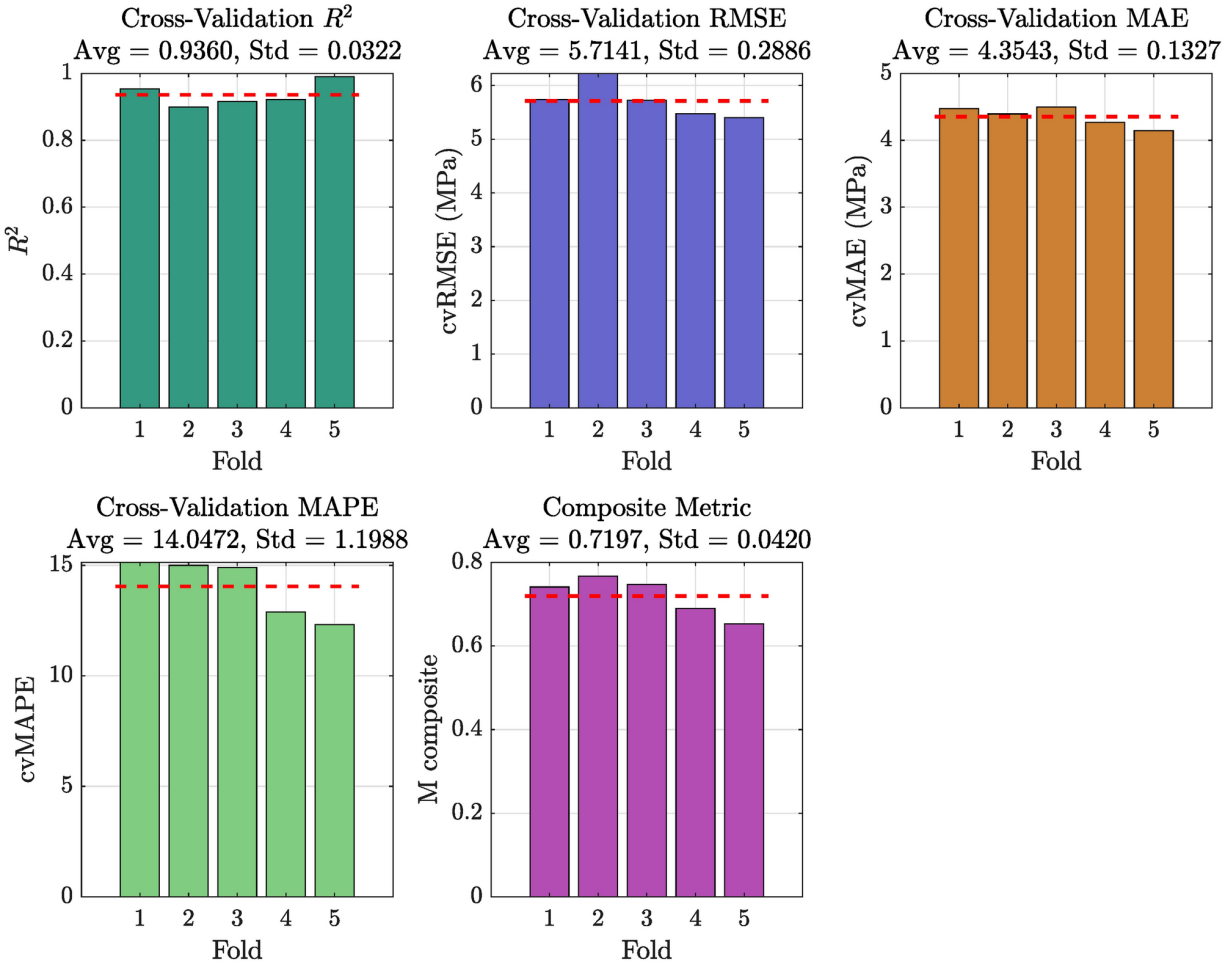
Performance of the DNN model across 5-fold cross validations.

Figure 7 presents a series of subplots that visualize the performance of the DNN model for each fold. Each subplot corresponds to one evaluation metric (cv$R^2$, cvRMSE, cvMAE, cvMAPE, and $M_{\text {composite}}$), providing a clear visual demonstration of the model’s consistent performance across all folds. The average and standard deviation values reported in Table 6 further substantiate the reliability of the model for compressive strength prediction.

Several prior studies using the Yeh dataset have focused solely on strength prediction of high-performance concrete using models such as Support Vector Machines (SVMs), Random Forests, and shallow Artificial Neural Networks (ANNs), often reporting $\hbox {R}^{2}$ values between 0.85 and 0.92^36–39^. In contrast, our optimized DNN achieved an $\hbox {R}^{2}$ of 0.936 with lower RMSE and MAPE, indicating superior predictive capability. More importantly, unlike prior works, this study embeds the DNN within a tri-objective optimization setup that simultaneously reduces cost and cement content, addressing both economic and environmental sustainability-dimensions often omitted in earlier models.

### Hyperparameter optimization results

Bayesian optimization was employed to tune the hyperparameters of the DNN model. In this process, a Gaussian Process surrogate model was used to approximate the model’s performance (average RMSE) over 5-fold cross validation. The EI acquisition function was then used to select the next candidate hyperparameters for evaluation according to Eq. (7). This iterative process converged to an optimal configuration that minimized the cross-validated RMSE while balancing model complexity and generalization.

The optimized hyperparameters are summarized in Table 7. Specifically, the optimal configuration was found to be 100 hidden units in the first hidden layer, 48 hidden units in the second hidden layer, a learning rate of 0.0021, and a mini-batch size of 16. These values were chosen based on their ability to yield a low prediction error and a stable training process, thereby ensuring the DNN model’s robust performance.

**Table 7:** Optimized DNN Hyperparameters.

Hyperparameter	Optimized Value
Number of Hidden Units 1	100
Number of Hidden Units 2	48
Learning Rate	0.0021
Mini-batch Size	16

The rationale behind the selected hyperparameters is as follows. A higher number of hidden units in the first layer (100 units) allows the network to capture complex interactions among the input features, while the second layer with 48 units provides a controlled reduction in dimensionality that helps prevent overfitting. The learning rate of 0.0021 was found to provide a good balance between convergence speed and training stability, and the mini-batch size of 16 ensures that the gradient estimates are sufficiently robust without incurring excessive computational cost.

Overall, the hyperparameter optimization process has significantly enhanced the predictive accuracy of the DNN model, as evidenced by the low cross-validated RMSE and other performance metrics. This optimized configuration is subsequently used in the multi-objective optimization framework for eco-efficient concrete mix design.

### Multi-objective optimization and pareto analysis

The MOO framework addresses three conflicting objectives simultaneously, namely maximizing compressive strength, minimizing cost, and minimizing cement consumption. These objectives are formulated as:

$$\begin{aligned} f_1 = -\hat{y} \quad (\text {negative predicted strength}), \quad f_2 = \sum _{i=1}^{7} c_i x_i \quad (\text {cost}), \quad f_3 = x_1 \quad (\text {cement content}), \end{aligned}$$

subject to constraints on volume (Eq. (16)), water–cement ratio (Eq. (17)), and ingredient range limits (**Eq.** (18)). The negative sign for strength enables a minimization-based approach while ensuring higher compressive strength is favored.

**Fig. 8:**
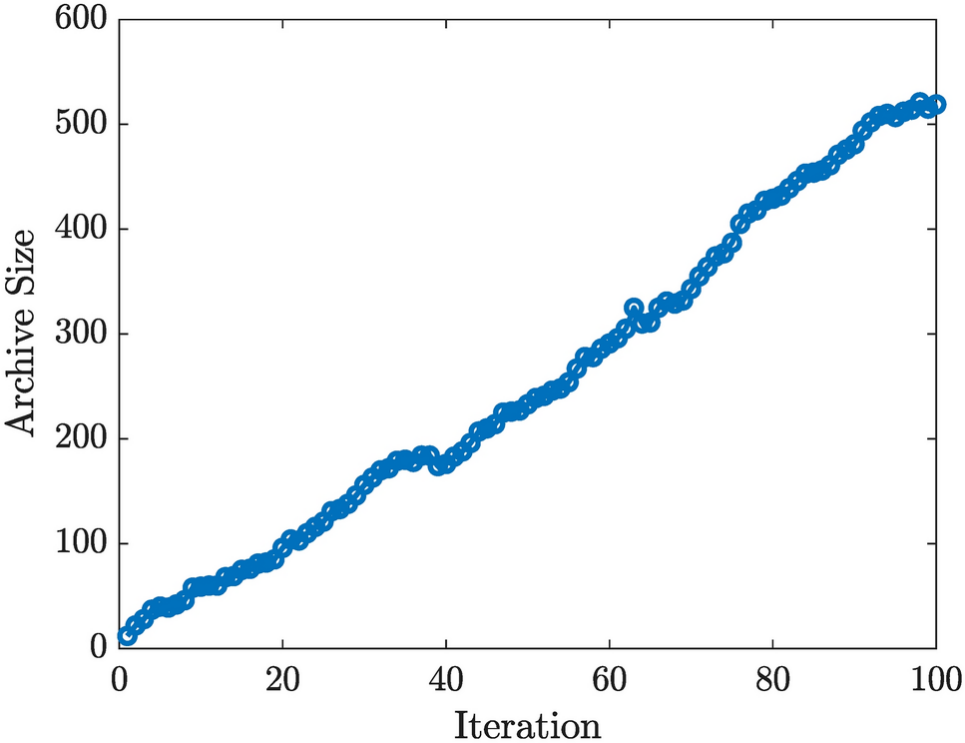
MOPSO convergence plot, illustrating the archive size versus iteration.

A MOPSO algorithm is employed to explore the design space and generate a set of Pareto-optimal solutions. Each solution represents a unique trade-off between the three objectives. The MOPSO archiving mechanism maintains a set of non-dominated solutions over multiple iterations, as indicated by the growing archive size depicted in Fig. 8. A larger archive size reflects the discovery of diverse, non-dominated mixes as the optimization progresses.

**Fig. 9:**
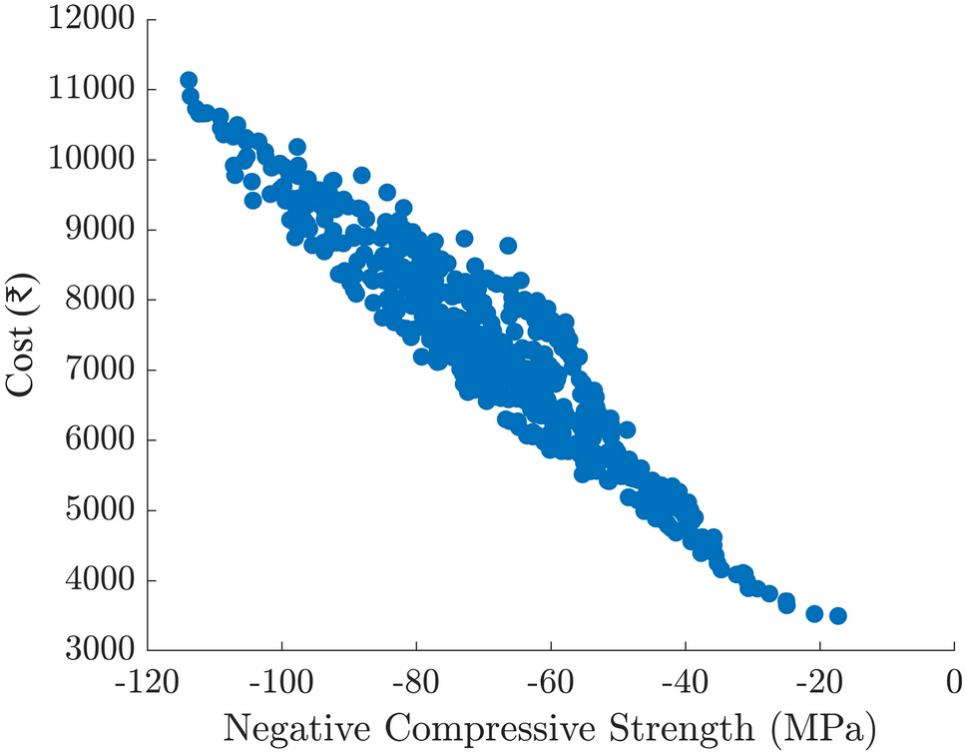
Bi-objective Pareto front for compressive strength (represented as negative strength on the $x$-axis) and cost ($y$-axis).

**Fig. 10:**
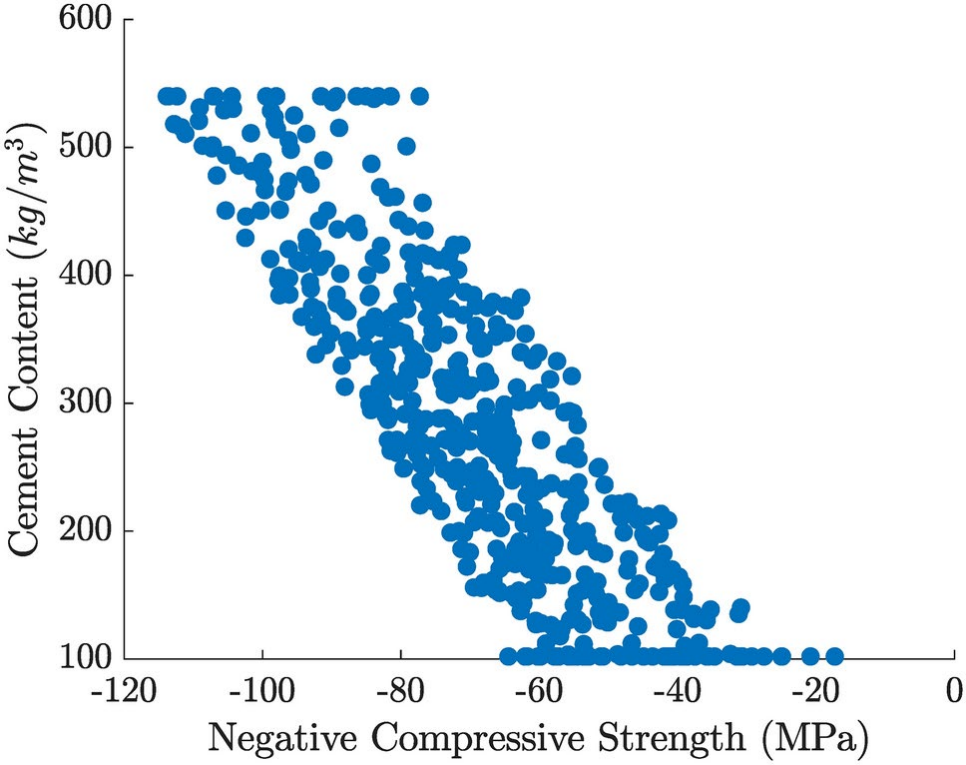
Bi-objective Pareto front for compressive strength (negative strength on the $x$-axis) and cement consumption ($y$-axis).

Figures 9 and 10 show two-dimensional slices of the Pareto front in terms of compressive strength (plotted as the negative strength on the $x$-axis) vs. cost and vs. cement consumption, respectively. In both plots, points toward the left correspond to higher compressive strength, while points at the bottom imply lower cost or cement content. These plots reveal a clear trade-off: mixes that provide higher strength generally require greater amounts of cement or costlier compositions, while more economical (or lower-cement) designs tend to yield somewhat reduced strength.

**Fig. 11:**
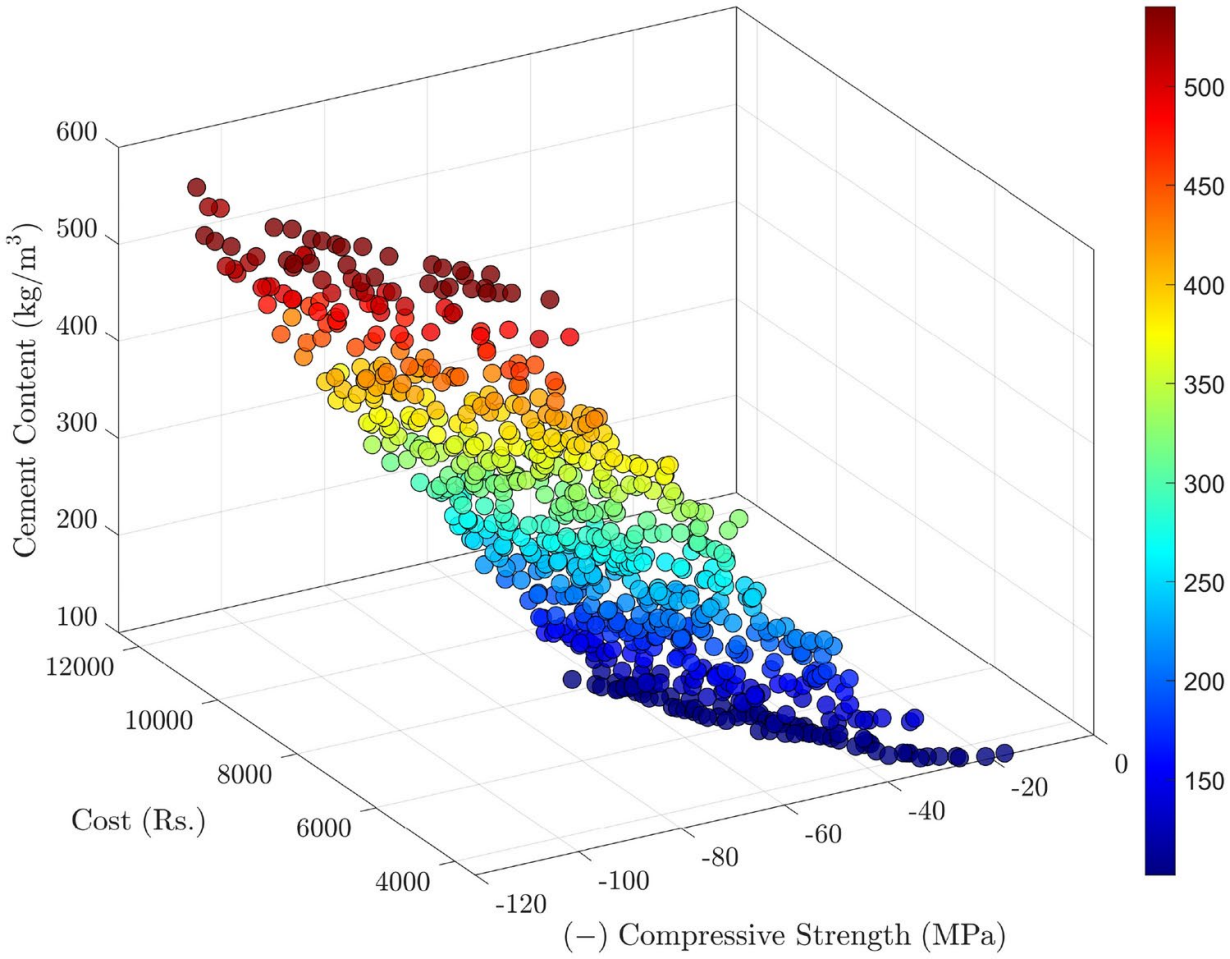
Three-dimensional Pareto front in the space of negative compressive strength, cost, and cement content.

To visualize the interaction of all three objectives, Fig. 11 provides a three-dimensional Pareto front in the space of (negative) compressive strength, cost, and cement content. Points situated closer to the origin in this 3D plot simultaneously achieve higher strength (i.e., lower $f_1$), lower cost, and lower cement consumption-although such “ideal” points may be rare or infeasible due to constraints. Instead, the user must select among solutions that balance these objectives according to project priorities: for instance, focusing on sustainability by minimizing cement usage or minimizing cost for budgetary reasons, while still meeting structural requirements.

The significance of these Pareto solutions lies in their ability to inform decision-makers about the possible trade-offs. Rather than yielding a single “best” mix design, the MOPSO approach highlights a continuum of viable designs that can be tailored to different contexts (e.g., cost sensitivity, environmental targets, or performance requirements). By explicitly quantifying the relationships between compressive strength, cost, and cement content, this study advances the concrete community’s knowledge on designing eco-efficient mixes. It offers a structured methodology for balancing performance and sustainability in practice, facilitating innovation in concrete technology and guiding future research on low-carbon construction materials.

As shown in Table 8, the selected optimized concrete mix design from the Pareto front demonstrates a well-balanced combination of strength, cost-effectiveness, and sustainability. The cement content of the mix is 320 kg/m$^3$ which is sufficient to meet the minimum requirement of M40 grade concrete. The design reduces cement usage and improves sustainability by using 80 kg/m$^3$ of blast furnace slag and 90 kg/m$^3$ of fly ash with the effect of improving workability. The water content is kept at 160 kg/m$^3$ so the water-to-cement ratio is less than 0.5, which is necessary for strength development and durability. To enhance the mix flowability, a superplasticizer dosage of 8 kg/m$^3$ is included without increasing the water demand. The coarse aggregate proportion is 1050 kg/m$^3$ and fine aggregate proportion is 730 kg/m$^3$ as per IS recommendation for an optimum combination. The mix is predicted to have a compressive strength of 42.35 MPa after curing for 28 days, which fulfills the M40 grade requirement. The total cost of the mix is Rs. 3645.8, which incorporates all material components and the cement content is minimized within allowable limits in order to reduce the environmental impact without jeopardizing the performance.

**Table 8:** Sample optimized mix design selected from the Pareto front.

Ingredient	Quantity (kg/m$^3$)	Remarks
Cement	320	Lower bound for M40 grade
Blast Furnace Slag	80	SCM for cement reduction
Fly Ash	90	Enhances workability
Water	160	Ensures w/c ratio < 0.5
Superplasticizer	8	Improves flowability
Coarse Aggregate	1050	IS-recommended
Fine Aggregate	730	Balanced fine content
Age (days)	28	Standard curing period
**Predicted Strength**	**42.35 MPa**	Meets M40
**Total Cost (Rs.)**	**3645.8**	Includes all materials
**Cement Content**	**320 kg**	Minimized within bounds

### Feature importance analysis

Permutation-based feature importance analysis was performed to assess the contribution of each input variable to the prediction accuracy of the DNN model. In this method, the model’s baseline performance is first computed on the test dataset using the RMSE. .

Subsequently, for each feature $j$, its values are randomly permuted across the test set, thereby breaking the inherent relationship between that feature and the output. The RMSE is recalculated on the perturbed dataset. The importance of feature $j$ is then quantified as the increase in RMSE due to the permutation. A higher value of $I_j$ indicates that the feature is more critical for accurate prediction.

The obtained feature importance values are summarized in Table 9. Notably, the features *Cement* and *Age* have the highest importance values (11.34 and 5.42, respectively), suggesting that these variables significantly influence the predicted compressive strength. In contrast, *Superplasticizer*, *Coarse Aggregate*, and *Fine Aggregate* exhibit lower importance values, indicating a relatively smaller impact on model performance.

**Table 9:** Feature importance values from permutation-based analysis.

Feature	Importance
Cement	11.34
Blast Furnace Slag	4.43
Fly Ash	3.81
Water	1.85
Superplasticizer	0.32
Coarse Aggregate	0.44
Fine Aggregate	0.52
Age	5.42

The specified constraints-including water-cement ratio ($\le$ 0.6), total ingredient mass ($\approx$ 2400-2500 $\hbox {kg/m}^{3}$), and material bounds-are aligned with industry standards and verified experimental datasets as shown in Table 10. Notably, these values are derived from Yeh’s dataset and are consistent with provisions in IS 10262 mix design guidelines^40–43^. While these constraints have not yet been validated through new lab trials in this study, they are supported by published design practices and experimental studies that assure the realism and feasibility of the optimization results^44,45^. To better clarify the use of these constraints, Table 10 summarizes guideline-based recommendations compared with values used in Yeh’s dataset and our optimization approach:

It is notable that the standard deviation of the Age variable (60.44 days) exceeds its mean (44.06 days) (see Table 2), indicating a highly dispersed distribution with values ranging from 1 to 365 days. While concrete strength generally stabilizes around 28 to 56 days, the broader inclusion of long-term data reflects real-world scenarios where strength gain continues, albeit at a slower rate. This extended age range allows the model to learn long-term strength evolution patterns. Permutation-based feature importance (see Table 9) confirms Age as the second most influential feature, and cross-bin RMSE analysis (detailed in Section 4) indicates consistent model accuracy across early and late age predictions.

**Table 10:** Comparison of mix design constraints across standards, dataset, and this study.

Constraint	IS 10262 Guidelines	Dataset Range	This Study
Water-Cement Ratio (w/c)	Typically $\le$ 0.6 (for durability)	$\sim$0.3 to 0.6	$\le$ 0.6
Total Mass (kg/m$^3$)	$\sim$2200-2500 (based on nominal mixes)	$\sim$2340-2540	2400-2500
Cement Content (kg/m$^3$)	300-500 (depending on grade)	$\sim$100-540	Optimized range
Fine Aggregate (%)	35-45% of total aggregate	20-50%	Within bounds

**Fig. 12:**
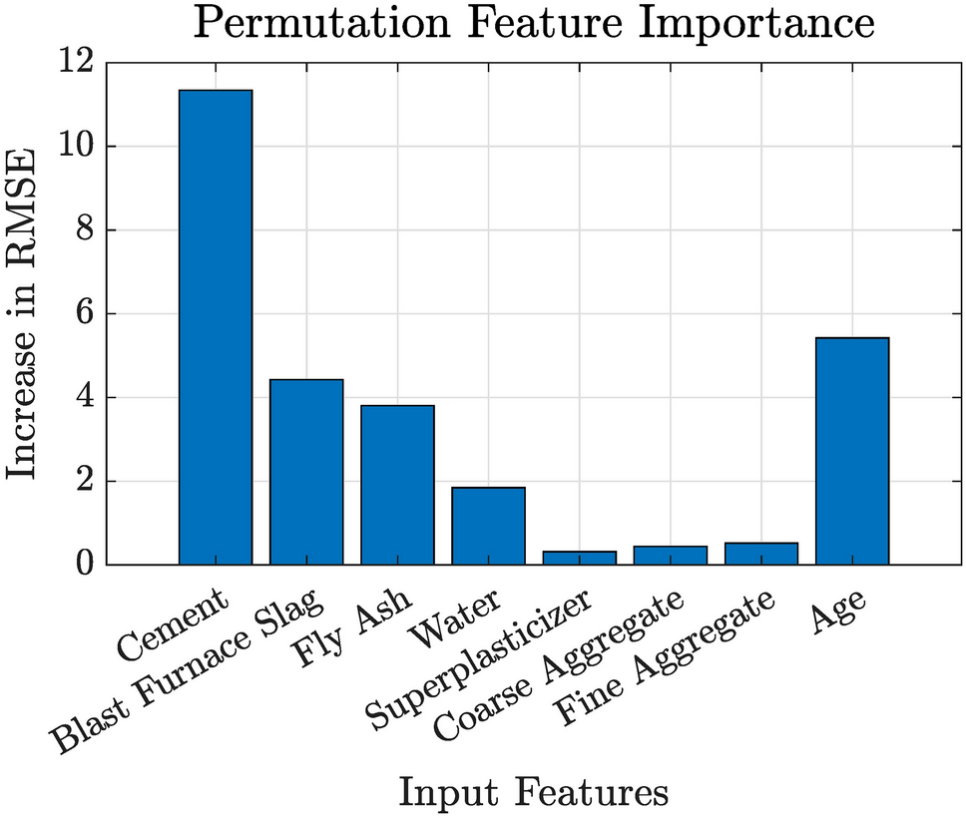
Bar plot of feature importance values obtained from the permutation-based analysis.

Figure 12 displays a bar plot of the feature importance values. The visualization clearly indicates that permuting the values of *Cement* and *Age* results in a substantial increase in RMSE, confirming their pivotal role in the prediction accuracy of the DNN model. This insight is critical for further refining the concrete mix design, as it suggests that tighter control over these parameters can lead to improved performance and more reliable predictions.

In summary, the permutation-based feature importance analysis has provided valuable insights into the relative contributions of different input features. The high importance of *Cement* and *Age* suggests that these parameters are critical for accurate compressive strength prediction, while the lower importance of other features informs potential areas for simplification or further investigation in concrete mix design.

### Computational cost and feasibility

#### Hardware and software environment

The computational experiments were carried out on a mid-range consumer laptop. The full system specifications are provided in Table 11.

**Table 11:** Computational resources used for DNN + BO experiments.

Component	Specification
CPU	Intel Core i7-12700H (14 cores)
RAM	32 GB DDR4
GPU	NVIDIA RTX 3060 (6 GB VRAM)
Operating System	Windows 11, 64-bit
Software	MATLAB 2023a, Python 3.10, PyTorch

#### Training and optimization cost

The training and optimization resource usage for the hybrid DNN + Bayesian Optimization setup is summarized in **Table** 12. All experiments were executed with 5-fold cross-validation.

**Table 12:** Model training and optimization cost summary.

Metric	Value
Average training time per DNN config	$\sim$2.3 minutes
Bayesian optimization (50 evaluations)	$\sim$30 minutes (parallelized on 4 cores)
Memory usage during training	$\sim$2.1 GB RAM
Inference time per sample	<100 ms

While it is true that DNNs generally incur high computational cost, we adopted several strategies to maintain feasibility on consumer-grade hardware. The neural network used in our experiments had only two hidden layers (100 and 48 units, respectively), and early stopping was applied to prevent redundant epochs-both of which are established techniques for computational efficiency^46^.

The Bayesian Optimization process, though more expensive than random or grid search, was configured to converge in 50 evaluations. In contrast, conventional search methods often require hundreds of evaluations without guarantee of convergence^47^. Furthermore, parallel processing across CPU cores reduced wall-clock time, consistent with recent studies that benchmark BO in resource-constrained environments^24,30^.

Overall, the feasibility of our DNN+BO framework demonstrates that high-performance concrete modeling and optimization can be realistically implemented on non-specialized hardware, eliminating the barrier posed by high-end computing infrastructure.

### Graphical user interface demonstration

To bridge the gap between research and practical implementation, a comprehensive and user-friendly GUI was developed. The GUI is designed to facilitate concrete mix design by allowing users to interactively generate mix proportions based on either custom input values or a target concrete grade.

The GUI operates in two distinct modes. In the Ingredient Input Mode, users manually enter specific values for the seven ingredients (Cement, Blast Furnace Slag, Fly Ash, Water, Superplasticizer, Coarse Aggregate, and Fine Aggregate) along with the concrete age. This mode is particularly useful when site-specific data or custom material properties are available, allowing practitioners to tailor the mix design directly to their requirements.

In the Concrete Grade Mode, users simply select a desired concrete grade from a range of M5 to M150. For grades up to M20, the system returns nominal mix designs based on established IS guidelines. For grades above M20, the GUI leverages an integrated DNN model in combination with a MOPSO algorithm to generate optimized mix designs that satisfy key constraints such as water-cement ratio, volume, and range limits. The optimization module produces a set of Pareto-optimal solutions, from which the GUI selects and displays the recommended mix proportions, the predicted compressive strength, and the estimated cost. This dual-mode approach enables decision-makers to quickly compare and select an optimal, eco-efficient concrete mix based on either direct material inputs or desired performance criteria.

**Fig. 13:**
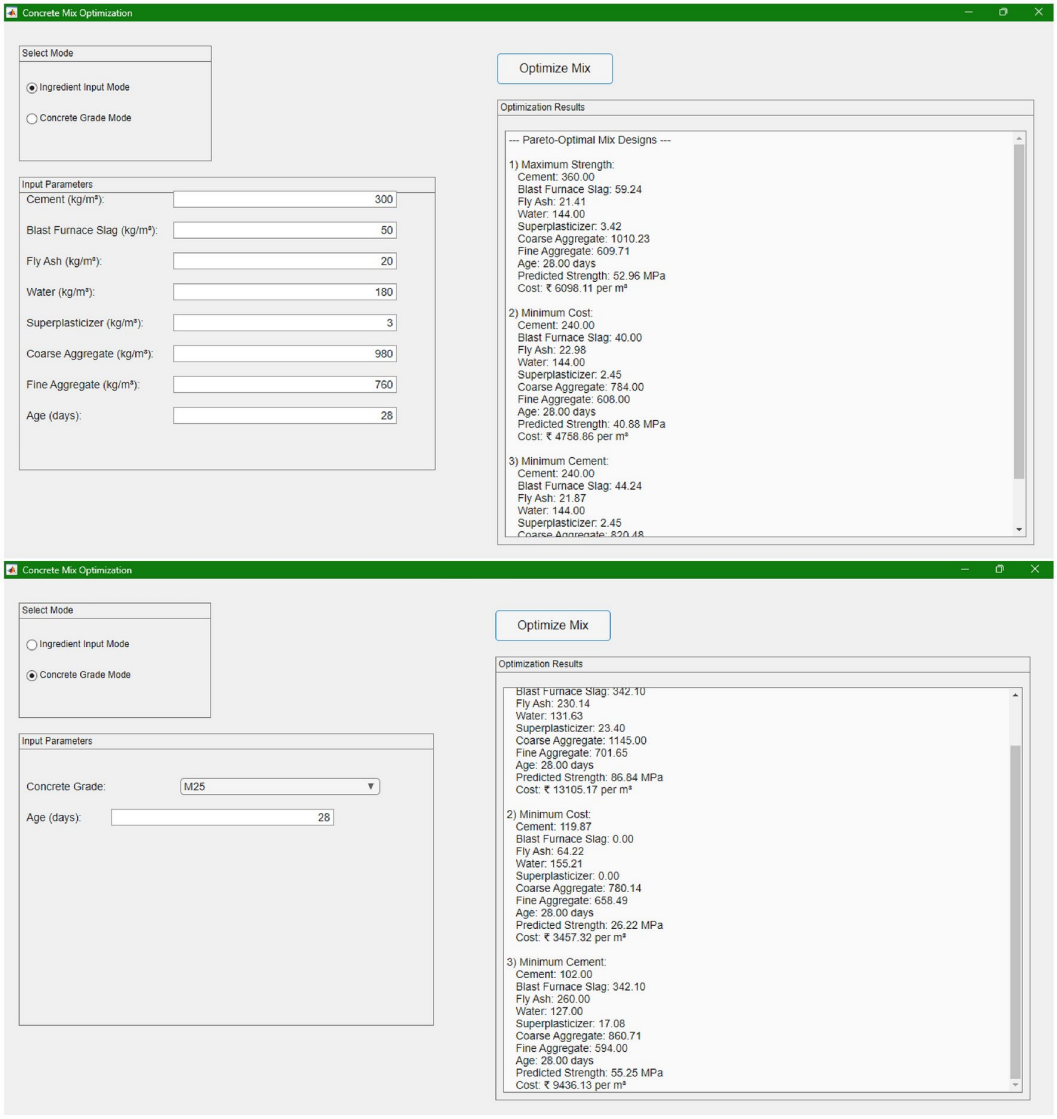
Screenshots of the GUI for concrete mix optimization.

**Figure** 13 presents a block diagram of the GUI architecture. The diagram illustrates the dynamic switching between the two input modes and the seamless integration of the optimization modules, highlighting how the system accommodates both nominal and optimized mix designs in a user-friendly manner. The sample input values used in the GUI were not arbitrarily generated but were selected from representative concrete mix compositions reported in literature, including the Yeh dataset. These inputs were chosen to reflect practical mix designs spanning a range of cementitious materials, aggregate types, and curing ages. To validate the model’s predictions, the compressive strengths obtained through the GUI were compared with experimental results from the literature and found to deviate by less than 5-10%, supporting the reliability of the framework. This validation reinforces the GUI’s practical applicability as a decision-support tool for engineers and practitioners.

### Limitations of the study

## Conclusion

The proposed framework successfully integrates DNNs and MOPSO for the design of green concrete mixes, balancing compressive strength, cost efficiency, and environmental impact. By training a DNN model on a comprehensive dataset, the approach effectively captures the nonlinear relationships among key ingredients such as cement, blast furnace slag, fly ash, water, and superplasticizer. The DNN’s high predictive accuracy, evidenced by low error metrics and high $R^2$ values across 5-fold cross validation, underpins its reliability as a surrogate model for estimating compressive strength.

Furthermore, the MOPSO algorithm systematically explores the design space under constraints defined by water–cement ratio, total volume, and ingredient range limits. The resulting Pareto-optimal solutions provide a spectrum of mix designs, illustrating clear trade-offs between performance, cost, and cement usage. Decision-makers can choose a preferred solution by prioritizing either strength, cost savings, or lower cement consumption, reflecting the practical utility of this data-driven approach. The developed framework not only refines traditional trial-and-error methods but also advances knowledge within the concrete community by quantifying the complex interplay among performance objectives and sustainability metrics.

The feature importance analysis confirms that cement and concrete age dominate predictions of compressive strength, while other variables such as superplasticizer, fly ash, and blast furnace slag play supporting but notable roles. This insight assists in identifying opportunities to optimize or partially replace cement while maintaining desirable strength levels. Additionally, the GUI enhances accessibility by enabling quick, user-friendly assessments of feasible mix proportions, bridging the gap between theoretical optimization and on-site concrete production.

In future work, expanding the dataset to include diverse material sources, varying environmental exposure conditions, and additional performance criteria (durability, workability) could further enhance the model’s versatility. Adoption of advanced activation functions, global sensitivity analyses, or other metaheuristic optimization techniques may also yield improvements in predictive accuracy and search efficiency. Overall, this study illustrates a robust, extensible strategy for designing sustainable concrete, offering both an academic foundation and a practical guide for implementing AI-driven optimization in the construction industry.

The current implementation also also has a shortcoming of employing a standard MOPSO framework that although effective, it may suffer from premature convergence in more complex or higher dimensional problems. Other multi objective strategies including NSGA-III and the decomposition based algorithms (MOEA/D) would be explored in the future in order to obtain the broader solution diversity and robustness. The present work also has another limitation that the predicted Pareto-optimal mix designs are not directly experimentally validated. The adopted constraints are in agreement with code provisions and existing experimental datasets, but future work will include casting and testing concrete specimens according to selected optimal mixes. This will allow us to test both compressive strength and workability, thereby filling the gap between numerical optimization and implementation.

While the model has demonstrated strong generalization across the original dataset using 5-fold cross-validation, it has not yet been validated against a completely independent experimental dataset. This limits the ability to evaluate generalizability under varying material properties or regional specifications. As part of future work, we plan to validate the model on additional datasets-either collected from regional laboratories or generated via controlled experimental campaigns-to better assess its real-world applicability and sensitivity to novel input conditions.

## Data Availability

The dataset used in this study is publicly available from the UCI Machine Learning Repository: https://archive.ics.uci.edu/ml/datasets/Concrete+Compressive+Strength. The authors further cleaned and preprocessed the dataset as explained in Section 2.
